# Machine learning of COVID-19 clinical data identifies population structures with therapeutic potential

**DOI:** 10.1016/j.isci.2022.104480

**Published:** 2022-05-31

**Authors:** David Greenwood, Thomas Taverner, Nicola J. Adderley, Malcolm James Price, Krishna Gokhale, Christopher Sainsbury, Suzy Gallier, Carly Welch, Elizabeth Sapey, Duncan Murray, Hilary Fanning, Simon Ball, Krishnarajah Nirantharakumar, Wayne Croft, Paul Moss

**Affiliations:** 1 Institute of Immunology and Immunotherapy, University of Birmingham, Birmingham, UK; 2 The Centre for Computational Biology, University of Birmingham, Birmingham, UK; 3 Institute of Applied Health Research, University of Birmingham, Birmingham, UK; 4 NIHR Birmingham Biomedical Research Centre, University Hospitals Birmingham NHS Foundation Trust and University of Birmingham, Birmingham, UK; 5 University Hospitals Birmingham NHS Foundation Trust, Birmingham, UK; 6 Institute of Inflammation and Ageing, University of Birmingham, Birmingham, UK; 7 Health Data Research, London, UK

**Keywords:** Viral microbiology, Bioinformatics, medical informatics

## Abstract

Clinical outcomes for patients with COVID-19 are heterogeneous and there is interest in defining subgroups for prognostic modeling and development of treatment algorithms. We obtained 28 demographic and laboratory variables in patients admitted to hospital with COVID-19. These comprised a training cohort (n = 6099) and two validation cohorts during the first and second waves of the pandemic (n = 996; n = 1011). Uniform manifold approximation and projection (UMAP) dimension reduction and Gaussian mixture model (GMM) analysis was used to define patient clusters. 29 clusters were defined in the training cohort and associated with markedly different mortality rates, which were predictive within confirmation datasets. Deconvolution of clinical features within clusters identified unexpected relationships between variables. Integration of large datasets using UMAP-assisted clustering can therefore identify patient subgroups with prognostic information and uncovers unexpected interactions between clinical variables. This application of machine learning represents a powerful approach for delineating disease pathogenesis and potential therapeutic interventions.

## Highlights

• UMAP-assisted clustering can define subgroups of acute COVID-19 patients
• These clusters could be validated in independent patient cohorts
• Cluster assignation on the day of admission was predictive of 28-day mortality
• Clusters uncover clinical interactions that may to guide treatment approaches

## Graphical abstract

**Figure:**
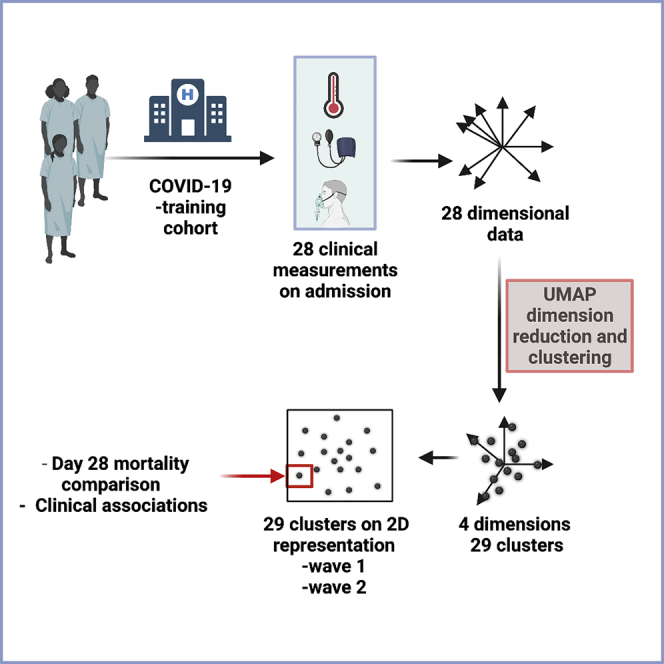

## Introduction

The COVID-19 pandemic has led to >4.6 million deaths to date, but the clinical outcome after primary infection is heterogeneous and approaches to predict outcome within individual patients are of value. Several demographic features increase the mortality risk, including age and comorbid conditions, and have been used to define clinical risk scores. The ISARIC4C mortality score is used widely and assesses nine demographic and laboratory values (Knight et al., 2020).

This clinical heterogeneity of COVID-19 has led to interest in further defining patient subgroups (Gutiérrez-Gutiérrez et al., 2021; Rodriguez et al., 2021), but although inclusion of more demographic and laboratory values can improve accuracy, the associated increase in data dimensionality is challenging for clustering algorithms. As such, integration of outputs from very high dimensional datasets usually requires either feature selection or dimensionality reduction. Although the latter approach loses some information, the associated compression and noise reduction greatly improves utility and is used commonly in biological assessments such as analysis of single-cell RNA sequencing data (Peyvandipour et al., 2020). The reduced dimensional outputs created through principal component analysis (PCA) are a linear combination of the input and struggle to capture complex nonlinearities (Jolliffe and Cadima, 2016). Nonlinear dimension reduction or manifold learning techniques such as Uniform Manifold Approximation and Projection (UMAP) (McInnes et al., 2018; Tang et al., 2016) are based on topological analysis and can define groups without prespecifying a target for the algorithm (Grollemund et al., 2020). The combination of UMAP dimensionality reduction and model-based clustering offer a powerful approach to unsupervised machine learning (Allaoui et al., 2020; Prabakaran et al., 2019).

To identify subgroups of patients with COVID-19, we obtained information on 28 clinical, demographic, and laboratory variables in a training cohort of 6099 patients on the day of their hospital admission with acute COVID-19 with outcomes followed for 28 days including inpatient and community mortality. UMAP dimensionality reduction and clustering were then applied to define clinical subgroups that predicted mortality and were confirmed in two large patient validation cohorts. Besides, it identified unexpected interrelationships between variables that could be valuable for guiding treatment pathways.

## Results

### Survival probability between cohorts and risk groups

**Figure 1:**
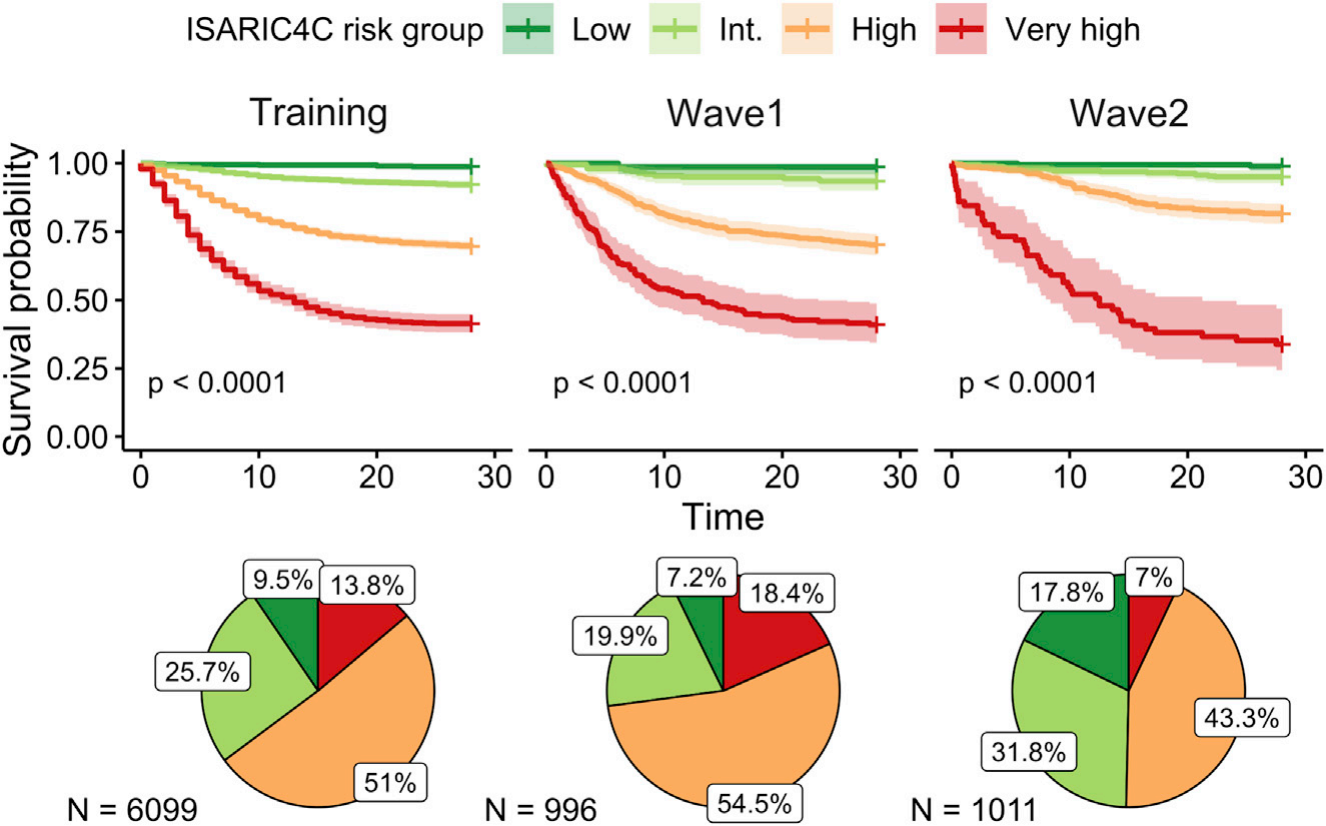
Survival probability by ISARIC4C risk group KM survival curves (95% CI) and composition of cohorts by risk group.

Significant differences in survival probability were observed between the cohorts (p < 0.0001). 28 days after admission, the survival probability in the training cohort was 74% (CI 73–75), whereas this was 71% in the wave 1 cohort (CI 69–74) and increased to 86% (CI 83–88) in wave 2. Patients within each ISARIC4C risk group exhibited markedly different outcomes within all cohorts (training p < 0.0001, wave 1 p < 0.0001, wave 2 p < 0.0001) (Figure 1). The relative frequency of ISARIC4C risk groups was comparable in the training and wave 1 cohorts, whereas a notable increase in the proportion of low and intermediate risk patients was seen in wave 2.

Low-risk patients had a consistently high survival probability of 99% (training CI 98–100; wave 1 CI 96–100; wave 2 CI 97–100). For the intermediate risk group, the survival probability was 92% (CI 91–94) in the training cohort, 93% (CI 90–97) in wave 1 and marginally higher in wave 2 at 95% (CI 93–97). High risk patients had a lower survival probability of 70% in both the training (CI 68–71) and wave 1 cohorts (CI 66–74), but this improved in wave 2 to 82% (CI 78–85). However, patients in the very high-risk group had a consistently poor prognosis in each cohort. The survival probability was 41% (CI 38–45) in the training cohort, 40% (CI 34–49) in wave 1, and 34% (CI 23–47) in wave 2.

Taken together, these data indicate that although overall prognosis improved for patients in wave 2, this was driven mainly by improvements in the outcome of intermediate and high-risk patients, with no apparent improvement for those in the very-high risk group.

### UMAP transformation and GMM clustering of clinical variables identifies distinct patient groups

**Figure 2:**
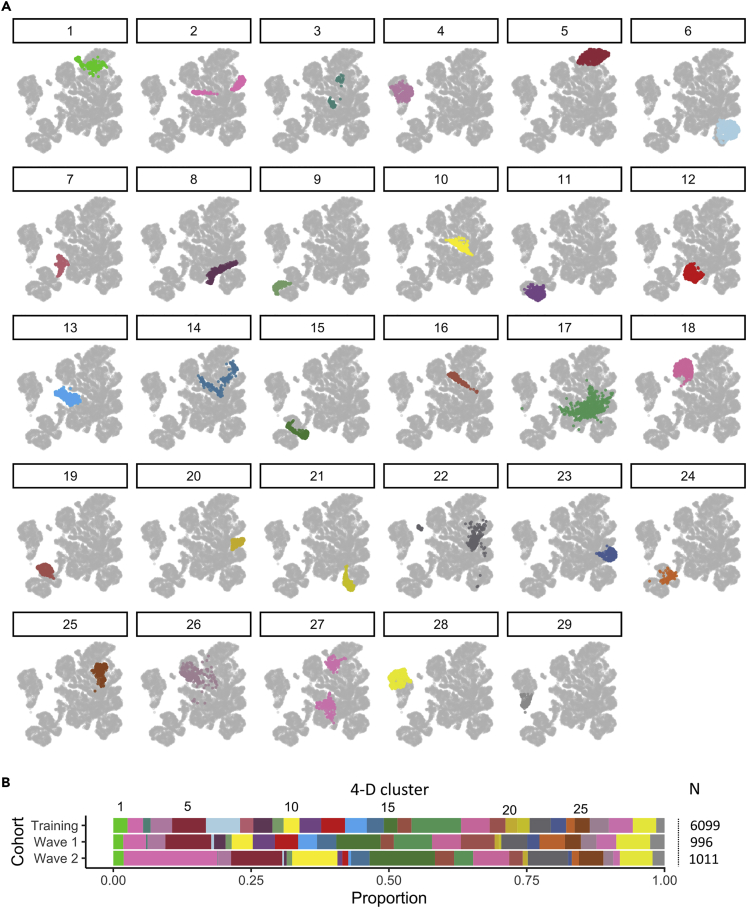
Visualization of patient clusters within the training cohort (A) Using a 4-D embedding as input, patients within the training cohort were classified into 29 clusters by fitting a GMM. Each 4-D cluster was then visualized by labeling the training cohort with colors on a 2-D embedding. (B) The fitted GMM was then applied to classify patients from waves one and two of the validation cohort into the same set of 4-D clusters. The proportion of patients assigned to each cluster was calculated within each cohort.

28 demographic and laboratory variables were obtained for each patient on the day of entry to hospital and used for inclusion within the UMAP analysis (Table 1). Patient subgroups were identified within the UMAP embedding using GMM clustering (Figure 2A).

**Table 1:** Characteristics of patients and clinical variables within the cohorts

Variable	Cohort			Adj. P value		
	Training	Wave 1	Wave 2	Wave 1 v Training	Wave 2 v Training	UMAP Input
Patient number	6099	996	1011			
Age (years) [IQR]	73.00 [58.00, 83.00]	70.00 [56.00, 83.00]	64.00 [50.00, 79.00]	0.04	0.01	✓
Male (%)	3361 (55)	564 (57)	525 (52)	0.97	0.16	✓
BMI (kg/m2) [IQR]	26.84 [23.31, 31.03]	27.78 [24.22, 32.73]	28.02 [23.95, 32.87]	0.01	0.01	✓
28-day deaths (%)	1570 (26)	284 (29)	146 (14)	0.22	0.01	
ISARIC4C Score [IQR]	10.00 [7.00, 13.00]	11.00 [8.00, 14.00]	9.00 [5.00, 11.00]			
Glasgow coma score [IQR]	15.00 [15.00, 15.00]	14.00 [9.00, 15.00]	15.00 [14.00, 15.00]			
Clinical frailty scale [IQR]	4.00 [2.00, 6.00]	4.00 [2.00, 6.00]	3.00 [2.00, 4.00]	0.02	0.01	✓
Cough (%)	4259 (70)	529 (58)	386 (38)	0.01	0.01	✓
Delirium (%)	1160 (20)	78 (9)	28 (3)	0.01	0.01	✓
Fever (%)	3212 (53)	454 (50)	303 (30)	0.41	0.01	✓
Cancer (%)	649 (11)	123 (12)	110 (11)	0.35	1.00	✓
COPD / Sleep Apnoea / Asthma (%)	1579 (26)	297 (30)	271 (27)	0.04	1.00	
CVD (%)	3033 (50)	531 (53)	293 (29)	0.12	0.01	✓
Dementia (%)	934 (15)	343 (34)	236 (23)	0.01	0.01	✓
Diabetes (any) (%)	1794 (29)	356 (36)	318 (32)	0.01	0.42	
Alt (U/L) [IQR]	24.00 [16.00, 41.00]	25.00 [16.00, 39.25]	25.00 [16.00, 41.00]	1.00	1.00	✓
Base excess [IQR]	0.20 [-2.00, 2.30]	-0.30 [-2.50, 2.00]	0.10 [-1.70, 1.70]	0.04	0.13	✓
Chest x-ray imaging (%)				0.01		
Appeared clear	1604 (29)	192 (24)				
Local consolidation	3226 (59)	229 (28)				
GGO / bilateral infiltration	637 (12)	389 (48)				
CRP (mg/L) [IQR]	73.00 [27.00, 146.00]	100.00 [43.00, 176.00]	55.00 [14.00, 118.50]	0.01	0.01	✓
Diastolic BP (mmHg) [IQR]	75.00 [66.00, 84.00]	75.00 [66.75, 84.00]	77.00 [69.00, 84.00]	1.00	0.01	✓
EGFR (mL/min) [IQR]	72.08 [47.41, 90.00]	68.00 [38.00, 90.00]	82.00 [58.00, 90.00]	0.01	0.01	✓
Fraction of inspired O2 (%) [IQR]	0.21 [0.21, 0.36]	0.21 [0.21, 0.21]	0.21 [0.21, 0.21]	0.01	0.01	✓
Hydrogen ion conc.(pH) [IQR]	7.41 [7.36, 7.45]	7.40 [7.36, 7.44]	7.40 [7.37, 7.44]	0.08	0.23	✓
Haemoglobin [IQR]	129.00 [114.00, 142.00]	129.00 [111.00, 144.00]	131.00 [117.00, 143.00]	1.00	0.16	✓
HCO3 [IQR]	24.30 [22.00, 26.50]	24.60 [21.85, 27.30]	24.60 [22.30, 26.70]	0.41	0.15	✓
Heart rate/min. [IQR]	90.00 [78.00, 104.00]	90.00 [79.00, 103.75]	88.00 [76.00, 102.00]	1.00	0.03	✓
Lactate (mmol/L) [IQR]	1.57 [1.13, 2.20]	1.69 [1.27, 2.29]	1.73 [1.36, 2.33]	0.01	0.01	✓
Lymphocytes (10*9/L) [IQR]	0.91 [0.62, 1.34]	0.92 [0.64, 1.30]	1.10 [0.73, 1.50]	1.00	0.01	✓
N:L ratio [IQR]	6.00 [3.47, 10.62]	6.00 [3.68, 10.49]	4.81 [2.86, 8.51]	1.00	0.01	✓
Neutrophils (10*9/L) [IQR]	5.63 [3.80, 8.50]	5.80 [4.05, 8.35]	5.20 [3.60, 7.97]	0.81	0.02	
O2 saturation (%) [IQR]	96.00 [93.00, 97.00]	96.00 [94.00, 97.00]	96.00 [94.00, 98.00]	0.03	0.01	✓
pCO2 [IQR]	4.63 [4.06, 5.38]	5.40 [4.70, 6.40]	5.40 [4.70, 6.10]	0.01	0.01	
Respiratory rate/min. [IQR]	20.00 [18.00, 25.00]	20.00 [18.00, 25.00]	19.00 [17.00, 23.00]	0.07	0.01	✓
Systolic BP (mmHg) [IQR]	130.00 [115.00, 144.00]	128.00 [114.00, 145.00]	127.00 [115.00, 145.00]	1.00	1.00	✓
Temperature (^°^C) [IQR]	37.10 [36.50, 38.00]	36.90 [36.20, 37.60]	36.60 [36.10, 37.30]	0.01	0.01	✓
Urea (mmol/L) [IQR]	7.50 [5.00, 12.00]	6.80 [4.60, 12.00]	5.70 [4.00, 8.80]	0.06	0.01	✓

Variables summarized by median (continuous) or count (categorical) by cohorts. Variables labeled with a ✓ were included as input into UMAP analysis.

Adj., adjusted; BMI, body mass index; BP, blood pressure; comp., complications; conc., concentration; COPD, chronic obstructive pulmonary disease; CRP, C-reactive protein; CVD, cardiovascular disease; EGFR, Estimated Glomerular Filtration Rate; GGO, ground glass occlusion; HCO3, bicarbonate; IQR, interquartile range; L, Liter; min., minute; mmol, Millimoles; N, number; N:L, Neutrophils:Lymphocytes; pCO2, partial pressure of carbon dioxide; U, units; umol, micromole.

A global estimate of intrinsic dimensionality indicated between two and six dimensions in the training cohort (Figure S4A). The FAMD scree plot indicated an “elbow” point at four or five dimensions (Figure S4B). Cluster silhouette was relatively stable with increasing dimensionality (Figure S4C). However, with >4 dimensions BIC comparisons showed greater variance (Figure S4D). From this, a UMAP embedding with four dimensions (‘4-D embedding’) was selected as input for clustering analysis.

The number of mixture components — K — was substantially higher when selected by BIC than by silhouette width (Figure S4E). As no clear optimal choice was apparent, several models were fitted, and the 28-day mortality rate was compared between clusters to ensure that the range of this value was comparable to that seen with the ISARIC4C score (Figure S4F). The manual BIC model resulted in the largest range of 28-day mortality (2–65%) where K = 29, in addition to comprising markedly fewer components than the optimal BIC model of K = 49, where the mortality range was 1–63%. In contrast, silhouette models resulted in substantially less diversity in the mortality range between clusters (e.g., 23–38% where K = 3 and 8–45% where K = 9). As such, K = 29 was chosen for onward analysis.

The final model — a GMM with 29 mixture components — was fitted to the training cohort using a 4-D embedding. The median number of patients per cluster was 175 (range 84–548). This model was then applied to patients within the validation datasets (Figure 2B) with comparable representation. Patient distribution within clusters was broadly similar between the training and validation cohorts. Strong cluster separation was observed using silhouette width analysis, although some clusters showed low levels of separation (Figure S5). In particular, clusters 17, 22, 26, and 14 each had consistently negative silhouette widths whereas clusters 27, 1, 24, and two had a negative silhouette only in the validation cohorts.

### Patient clusters defined from the training cohort are predictive of mortality rates within confirmation cohorts

**Figure 3:**
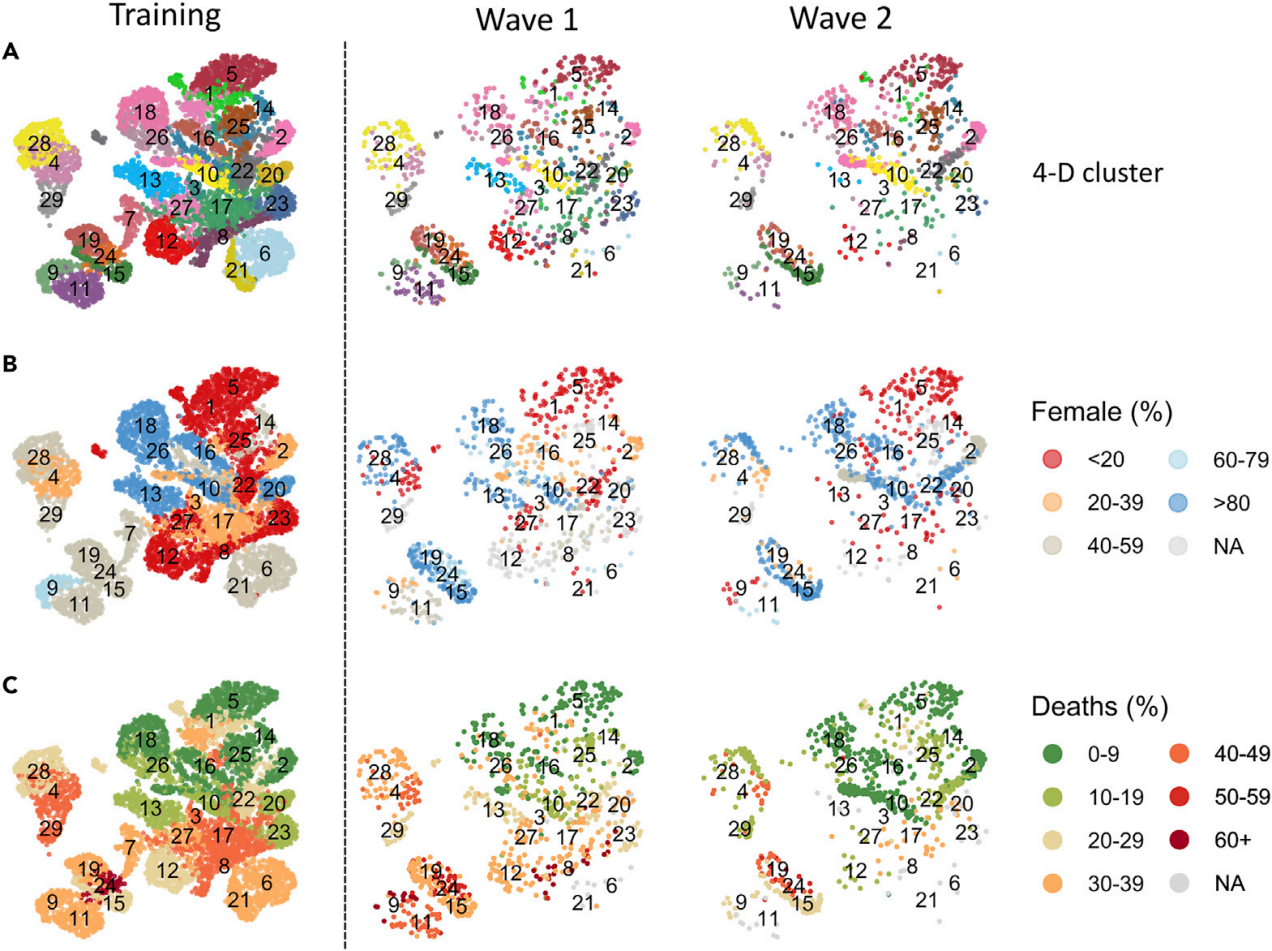
Sex allocation and Day-28 mortality within patient clusters (A) Representation of patient clusters in the three patient cohorts. (B) Sex distribution within each cluster. (C) Relative mortality at day 28 after admission within each cluster. Analysis was performed on clusters which comprised at least 10 patients.

2-D visualization of the 4-D clustering was used to display clusters across the three patient cohorts (Figure 3A). Allocation of sex to the clusters was investigated as this is a significant determinant of mortality. Relative distribution of sex varied between clusters and supported its importance of a key factor in clinical outcome (Figure 3B). We were next interested to assess their potential predictive power for subsequent mortality. The overall 28-day mortality rates for the training cohort and the wave 1 and 2 cohorts were 26%, 29, and 14%, respectively. Mortality at day 28 after admission varied markedly between clusters in the training dataset ranging from 2% within Cluster 18 to 65% in Cluster 24 (Figure 3C).

**Figure 4:**
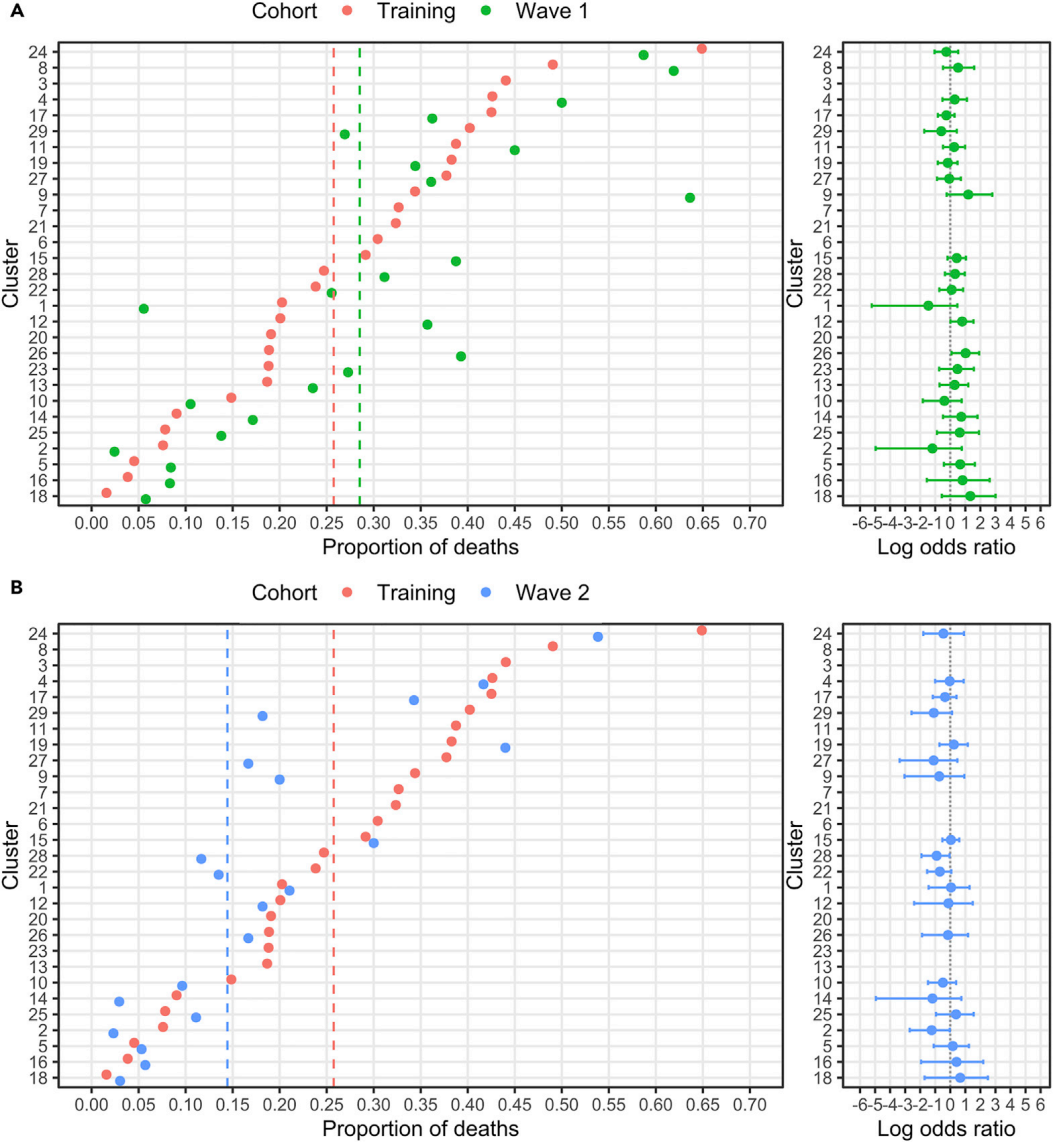
Day-28 mortality rate within patient clusters across study cohorts (A) The proportion of deaths by day 28 after admission within each cluster in the training and wave 1 cohorts. (B) The proportion of deaths by day 28 after admission within each cluster in the training and wave 2 cohorts. Dashed lines indicate the total proportion of deaths in each cohort. Clusters are ranked by mortality rate observed in the training cohort. Log odds ratios are calculated (95% CI, 1000 bootstraps) such that intervals centered on 0 indicate no difference in the odds of mortality between cohorts. Analysis was performed on clusters that comprised at least 10 patients.

These values were then compared to clinical outcomes for patients assigned to the equivalent cluster within the validation cohorts. Mortality prediction from cluster assignment modeled from the training dataset was found to be highly predictive for patients within the validation cohorts. Indeed, of the 24 clusters within the wave 1 validation cohort, only two had significantly different odds of mortality from the training cohort (Table 2). Specifically, Cluster 12 had a mortality rate of 36% compared to 20% in the training cohort (OR 2.2, p = 0.03), and Cluster 26 had values of 39 vs 19%, respectively (OR 2.8, p = 0.02) (Figure 4A). Cluster-associated mortality rates also showed a high degree of concordance between the wave 2 validation cohort and the training dataset (Figure 4B) with differences observed only for Cluster 2 (2 vs 8%; OR 0.3, p = 0.03) and Cluster 28 (12 vs 25%; OR 0.4, p = 0.04).

**Table 2:** Proportion of Day-28 deaths by cluster

		N patients			Day-28 deaths (%)			Odds ratio			p value		
Cluster	Rank	T	W1	W2	T	W1	W2	W1 v T	W2 v T	W2 v W1	W1 v T	W2 v T	W2 v W1
1	13	158	18	19	20	6	21	0.2	1.0	4.4	0.20	1.00	0.34
2	4	171	41	172	8	2	2	0.3	0.3	1.0	0.31	0.03	1.00
3	27	84	3	1	44								
4	26	237	32	24	43	50	42	1.3	1.0	0.7	0.45	1.00	0.60
5	3	375	83	94	5	9	5	1.9	1.2	0.6	0.17	0.78	0.55
6	17	378	5	3	30								
7	19	147	0	0	33								
8	28	210	21	5	49	62		1.7			0.36		
9	20	125	11	10	34	64	20	3.3	0.5	0.2	0.10	0.50	0.08
10	7	175	38	83	15	10	10	0.7	0.6	0.9	0.61	0.33	1.00
11	23	240	40	9	39	45		1.3			0.49		
12	12	264	42	11	20	36	18	2.2	0.9	0.4	0.03	1.00	0.47
13	8	241	34	5	19	24		1.3			0.49		
14	6	188	35	34	9	17	3	2.1	0.3	0.1	0.22	0.32	0.11
15	16	151	80	120	29	39	30	1.5	1.0	0.7	0.14	0.89	0.22
16	2	156	24	35	4	8	6	2.3	1.5	0.7	0.29	0.64	1.00
17	25	548	69	35	43	36	34	0.8	0.7	0.9	0.37	0.38	1.00
18	1	316	52	66	2	6	3	3.8	1.9	0.5	0.09	0.35	0.65
19	22	175	61	25	39	34	43	0.8	1.3	1.5	0.65	0.66	0.47
20	11	131	8	8	19								
21	18	136	4	1	32								
22	14	235	47	74	24	26	13	1.1	0.5	0.5	0.85	0.07	0.15
23	9	170	22	7	19	27		1.6			0.39		
24	29	94	46	13	65	59	54	0.8	0.6	0.8	0.58	0.54	0.76
25	5	166	29	45	8	14	11	1.9	1.5	0.8	0.29	0.55	0.73
26	10	212	28	18	19	39	17	2.8	0.9	0.3	0.02	1.00	0.19
27	21	265	36	12	38	36	16	0.9	0.3	0.4	1.00	0.22	0.29
28	15	259	61	60	25	31	12	1.4	0.4	0.3	0.33	0.04	0.01
29	24	92	26	22	40	27	18	0.6	0.3	0.6	0.26	0.08	0.51

The proportion of deaths by day 28 within each cluster in the training, wave 1 and wave 2 cohorts. Odds ratios are calculated with a Fisher’s test of significance. Clusters are ranked by the proportion of deaths observed within the training cohort.

N, number; T, training cohort; W1, wave 1 cohort; W2, wave 2 cohort.

These data show a high level of correspondence between the cluster-specific mortality rates in the training cohort and those seen in the validation cohorts indicating confidence that clustering can be generalized to external and temporally independent patient cohorts.

### Mortality rates within clusters reveal differential improvement in outcome between wave one and wave two of the COVID-19 pandemic

The period between waves one and two witnessed changes in the clinical management of COVID-19, and the mortality rate of 29% in wave 1 fell to 14% in wave 2. As such, we were interested to determine the utility of cluster modeling to assess relative improvements within different patient subgroups across this period.

Several differences in the demographic profile of patients between the two validation cohorts were apparent. Patients within wave 1 were an average 6 years older than those in wave 2 and had higher rates of comorbidity. Indeed, 53% had cardiovascular disease compared to 29% in wave 2 and rates of dementia or cancer also both fell during wave 2 (34 vs 23%; 12 vs 11%) (Table 1). In addition, the proportion of patients with diabetic complications halved to 5% in wave 2 (Table S1).

**Figure 5:**
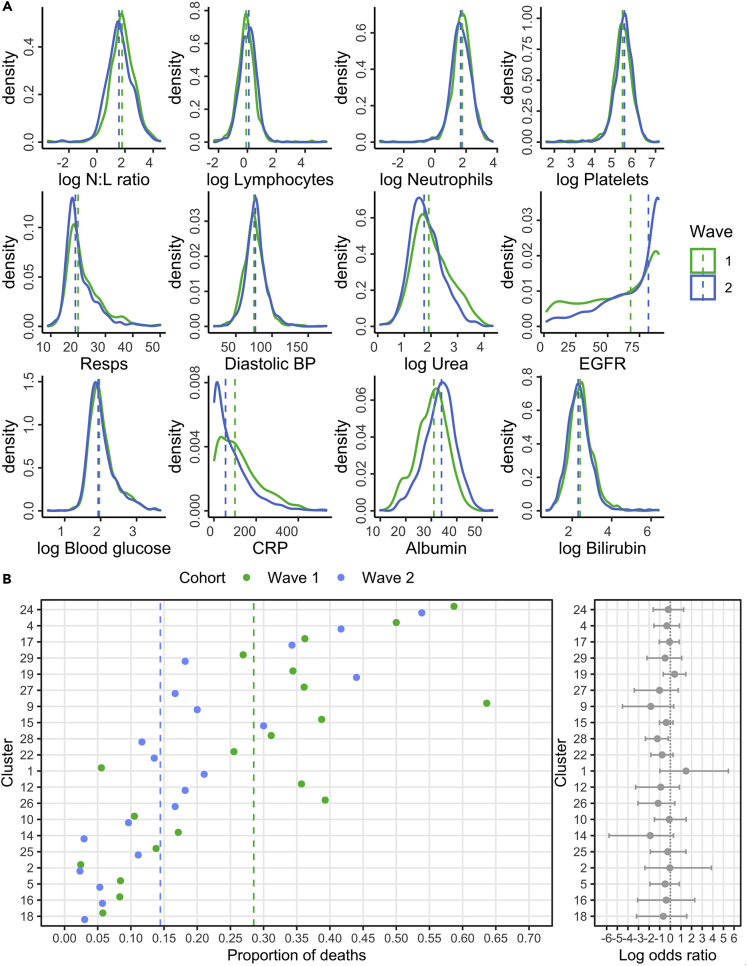
Distribution of clinical variables and cluster-associated mortality rates within waves one and two of the validation cohorts (A) Distribution of clinical variables in patients within the validation cohorts. Dashed lines indicate the median values. Missing values were excluded. (B) The proportion of deaths by day 28 after admission within the validation cohorts. Dashed lines indicate the total proportion of deaths. Clusters are ranked by mortality rate observed in the training cohort. Log odds ratios are calculated (95% CI, 1000 bootstraps) such that intervals centered on 0 indicate no difference in the odds of mortality between cohorts. Analysis was performed on clusters which comprised at least 10 patients.

Changes in the profile of laboratory variables were also apparent over time. For instance, the neutrophil:lymphocyte (N:L) ratio fell from 6.0 to 4.8, driven by an increase in lymphocyte count and less marked neutrophilia, whereas estimated glomerular filtration rate (EGFR) and albumin both showed improvements (68 vs. 82 mL/min; 31 g/L vs 34 g/L) (Figure 5A).

We next contrasted cluster-associated mortality rates for patients in wave 1 and wave 2, noting that these were managed in the same hospital. Comparison was possible for 20 clusters, of which, 17 showed a decrease in the odds of mortality, whereas two increased and Cluster 2 remained unchanged (Figure 5B). However, these changes were only significant for Cluster 28, which showed a 70% fall in the odds of mortality within wave 2 (OR 0.3, p = 0.01). Clusters with above average mortality showed little evidence of improvement such as clusters 4, 17, and 24 (OR 0.7, p = 0.6; 0.9, p = 1; OR 0.8, p = 0.76). Strikingly, the number of patients within Cluster 2, which was associated with a very low mortality rate, increased markedly from 4% to 17% between waves 1 and 2.

As such, these findings reveal that the fall in mortality rate in wave 2 was not uniform across clusters. There was a marked improvement in outcomes in the middle of the risk spectrum, whereas high-risk clusters continued to have a bad prognosis. Moreover, low risk patients remained highly likely to survive.

### Patient clusters reveal interactions between clinical variables that determine patient outcome

As patient clusters varied markedly in relation to 28-day mortality, we were next interested to determine the profile of clinical variables within each subgroup. In particular, the unsupervised nature of the analysis was thought likely to uncover interactions between variables that were not predictable before UMAP transformation and clustering.

**Figure 6:**
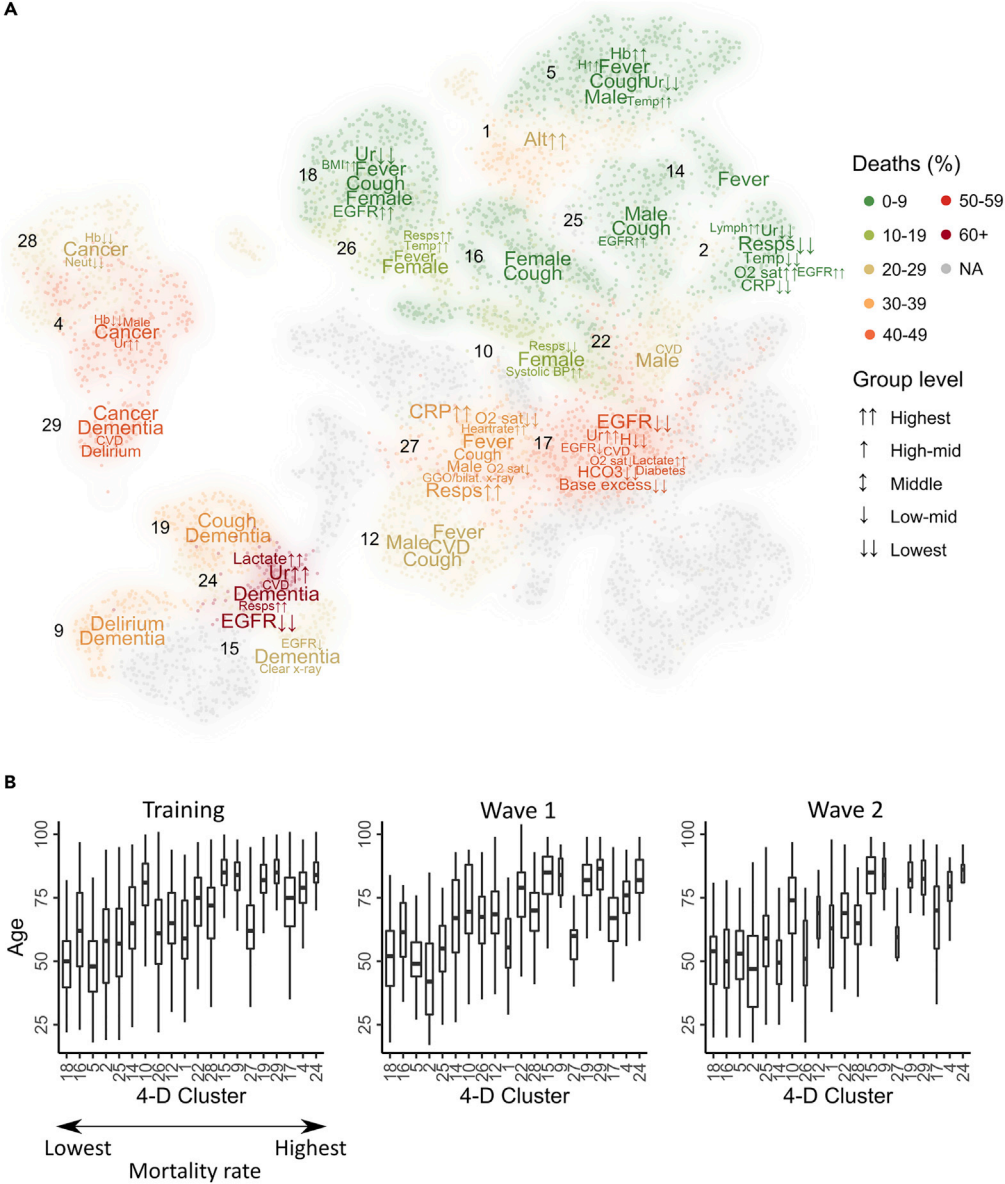
Cluster characterization reveals clinical variables associated with clusters (A) Clinical variables associated with patient clusters in the training cohort and at least one validation cohort (p = 0.001). Continuous variables were categorized, and Fisher’s tests were applied to each factor level. The strength of the association was used to construct a word cloud with larger words having a stronger association with a cluster in the training cohort. (B) Age distribution of patients within clusters. Boxes represent the median and IQR with whiskers extended to the lowest/highest datum within 1.5 IQR of the lower/upper quartile. Clusters are ordered along the x axis in relation to the increasing rate of associated 28-day mortality. Analysis was performed on clusters which comprised at least 10 patients in each cohort.

Variables that were significantly associated with each cluster were identified and those which could be confirmed in more than one cohort were presented as a word cloud on the UMAP plot (Figure 6A) where the size of the word was scaled according to strength of association with the cluster.

**Figure 7:**
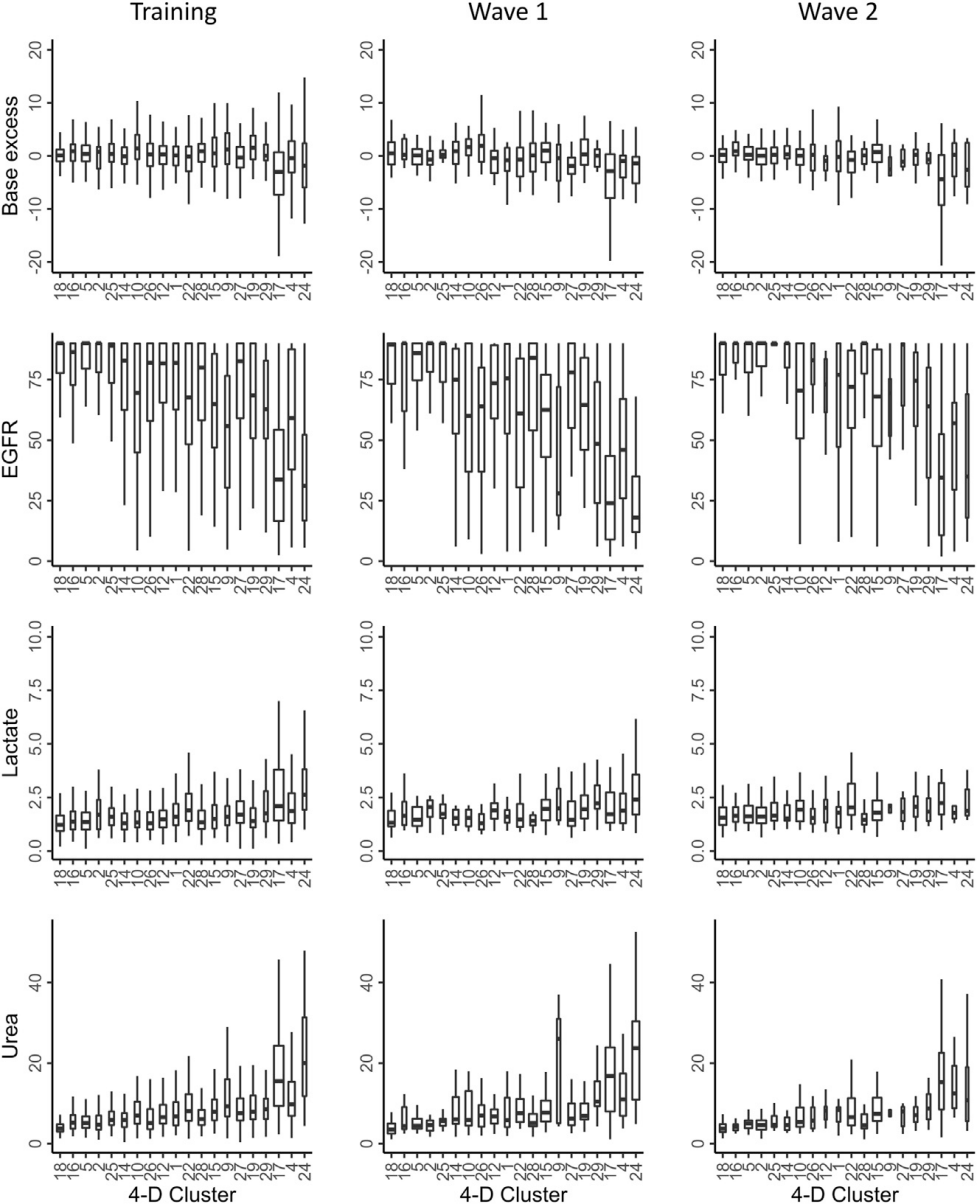
Distribution of individual clinical variables by cluster Example of the distribution of four clinical variables by cluster across the three patient cohorts. Base excess, EGFR, lactate, and urea are shown. Boxes represent the median and IQR with whiskers extended to the lowest or highest data within 1.5 IQR of the lower or upper quartiles. Clusters are ordered along the x axis in relation to the increasing rate of associated 28-day mortality. Missing observations were excluded, and the number of observations was used to scale the width of each box. Analysis was performed on clusters which comprised at least 10 patients in each cohort.

**Figure 8:**
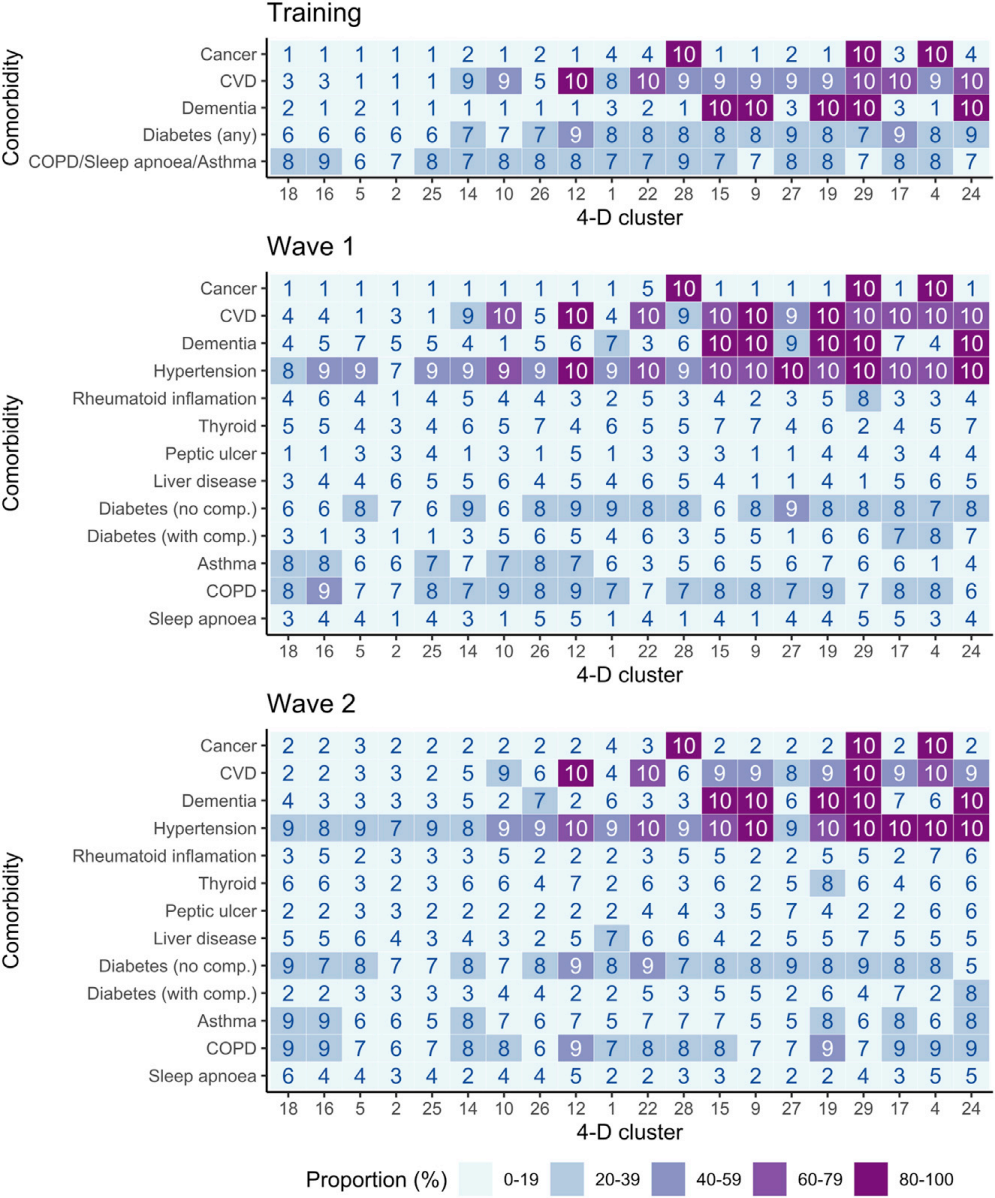
Proportion of patients with comorbidity within each cluster The proportion of patients diagnosed with a comorbidity by cluster is represented as a heatmap by decile. Clusters are ordered along the x axis in relation to the increasing rate of associated 28-day mortality. Analysis was performed on clusters which comprised at least 10 patients in each cohort.

Interesting relationships between variables were observed within the clusters. Increasing age correlated with the clinical severity of cluster-associated outcome, but it was noteworthy that this was not uniform (Figure 6B). Cluster 24, which showed the highest rate of mortality, included many patients with dementia and was associated with low EGFR and high levels of lactate and urea, suggesting a prognostic interaction between impaired renal function and metabolic acidosis (Figures 7 and 8). In contrast, Cluster 17 comprised somewhat younger patients with markedly low EGFR and high levels of lactate and urea.

Furthermore, this process of deconvolution of the determinants of clinical risk within clusters does not need to be limited to variables included within the UMAP transformation, allowing additional features associated with each cluster to be assessed in relation to prognostic importance. As an example, the association tests for patients within the Cluster 17 validation cohorts were repeated to include additional variables that were absent in the training cohort (Table 3) and revealed high potassium concentration (K+) as an additional feature of this cluster.

**Table 3:** Clinical variables associated with Cluster 17

Training	Wave 1	Wave 2
Base excess↓↓	CVD	Base excess↓↓
CRP↑↑	EGFR↓↓	**Breathlessness**
CVD	H↓↓	**Diabetes (with comp.)**
**Diabetes (any)**	**K↑↑**	EGFR↓
EGFR↓	pocFiO2↑↑	EGFR↓↓
EGFR↓↓	Ur↑↑	H↓↓
Frailty↕		HCO3↓↓
H↓↓		**Hypertension**
Hb↓↓		Lactate↑↑
HCO3↓↓		O2 sat↓
Lactate↑↑		Ur↑↑
O2 sat↓		
O2 sat↓↓		
pocFiO2↑↑		
Ur↑↑		

Clinical variables significantly associated with Cluster 17 by cohort (p = 0.001). Variables highlighted in bold were not included within UMAP analysis.

Comp., complications; CRP, C-reactive protein; CVD, cardiovascular disease; EGFR, estimated glomerular filtration rate; K, potassium; H, hydrogen ion concentration; Hb, hemoglobin; HCO3, bicarbonate; pocFiO2, fraction of inspired O2; O2 sat, O2 saturation; Ur, Urea.

## Discussion

Clinical practice is focused increasingly on defining and managing subgroups of patients to facilitate personalized approaches to therapy. Such a process is underway in conditions such as cancer where the use of genetic mutation testing has led to stratification of management (Middleton et al., 2020). However, across most conditions this process is not well advanced. The huge amount of demographic and clinical information that is acquired during patient assessment offers the opportunity to assess discrete subgroups of patients, but a major challenge has been the complexity of using such large datasets with the associated high-level dimensionality that they bring. Here, we used UMAP dimension reduction and GMM clustering to define subgroups of patients with acute COVID-19. We found that clusters were consistent between different populations, are predictive of subsequent mortality, and can be used to uncover unexpected relationships between clinical features.

We chose to use UMAP for dimensionality reduction; this uses topological data analysis and a cross validation optimization process to create a lower dimensional embedding, which retains much of the original data structure. This is an alternative approach to tSNE analysis but is scalable to larger datasets and more effectively captures local as well as global data structure (McInnes et al., 2018). A key advantage of UMAP is that the transformation can be applied to embed new observations into the same latent space, allowing it to be built into a model development pipeline. UMAP has been applied to a range of clinical and biological analyses, including stratification of patients with ALS (Grollemund et al., 2020), polygenic risk prediction (Sakaue et al., 2020), and single cell analyses (Becht et al., 2019).

Grollemund et al. (2020) demonstrated that UMAP is capable of stratifying patients into risk groups and used dimension reduction to develop a 1-year mortality prognostic model based on the distribution of patients on a 2-D embedding and comparison of groups based on coordinates on a grid. We extended this approach by dividing the embedding into subgroups through unsupervised clustering. UMAP preprocessing can also improve the results of clustering algorithms (Allaoui et al., 2020; Hozumi et al., 2021). The combination of risk-stratification and improved clustering thus makes UMAP-assisted clustering a powerful approach for the detection of novel clinical risk groups.

The default output from UMAP — a 2-D embedding — is ideal for visualization purposes. However, when applying UMAP for nonlinear dimension reduction, the choice is less clear. Our literature review did not indicate an established method to select dimensionality in this context. This decision will depend on both the complexity of the data and the ability of UMAP to represent topological structure on a lower-dimensional embedding. A qualitative approach was therefore undertaken, and a 4-D embedding was selected based on a global estimate of intrinsic dimensionality and assessment of the impact of dimensionality on the clustering model. Other parameters such as the number of nearest neighbors and minimum distance were fixed in our analysis.

Unsupervised clustering analysis was used to label patients by cluster given their distribution in UMAP space. BIC and silhouette width are validated methods for selecting the number of clusters and were adopted in this approach (Batool and Hennig, 2021; Gómez-Rubio, 2021; Keribin, 2000; Rousseeuw, 1987). Selecting the number of clusters is a widely debated topic (Chiang and Mirkin, 2010; Fu and Perry, 2020; Fujita et al., 2014; Gómez-Rubio, 2021). A useful property of a GMM is that it can be used to approximate any other probability density function, given a sufficient number of mixture components (Nguyen et al., 2020). This allows the model to fit clusters which follow a non-Gaussian distribution. However, in this context the model may require multiple mixture components to fit a single cluster. BIC will optimize the number of components to best fit the underlying data distribution rather than the number of distinct clusters. When the assumption is made that each component corresponds to a single cluster of patients, BIC may lead to overestimates in the true number of clusters (Baudry et al., 2010; Gómez-Rubio, 2021). Silhouette, on the other hand, measures the compactness within each cluster and the separation between them (Rousseeuw, 1987). Using silhouette to select the number of components therefore ensures the most distinct clustering configuration but can fail to give insight into clustering quality if cluster distributions require multiple components to be fitted (Gómez-Rubio, 2021).

In our analysis, the optimal BIC indicated 49 components but began to plateaux beyond 29. In contrast, the maximum silhouette corresponded to three components but showed a secondary peak with 9. This large discrepancy suggested BIC may have somewhat overestimated the number of clusters, whereas silhouette provided a relative underestimate. We therefore compared the diversity of 28-day mortality risk between clusters, to optimize the potential clinical value of the modeling and found that the model with 29 components resulted in the largest range (2–65%). As such, the decision to use BIC resulted in a model with a greater diversity of mortality risk between clusters, although some clusters had a negative silhouette width, indicative of relatively poor separation. Future work will investigate the use of an entropy criterion to combine components based on the loss of information (Baudry et al., 2010) and examine methods of determining groups from multiple survival curves (Villanueva et al., 2019). Other model-based clustering methods such as Hierarchical Density-based Spatial Clustering of Applications with Noise (Campello et al., 2015) and the use of Dirichlet processes can also be explored (Kottas, 2006).

As cluster labels are independent of outcome, unbiased comparisons can be made about the observations assigned to each subgroup. We chose to compare 28-day survival rates but other clinical outcomes, such as subsequent development of long-covid, could be compared without retraining the model. Model-based clustering allows validation of clusters in independent cohorts as the fitted model can be used to cluster new data. Indeed, we observed a concordance of results between the training and validation cohorts, which indicates that this model could potentially be used for analysis of any COVID-19 dataset provided the same predictors were available.

We applied the clustering approach to clinical variables taken on the day of hospital admission to predict subsequent mortality at day 28. This time point was chosen as most patients who die from acute COVID-19 succumb by 4 weeks after hospital entry, and it was felt that it would be challenging to extend prognostic modeling beyond this time. The associated mortality rates of individual patient clusters were very heterogeneous and ranged from 2% for the 316 patients in Cluster 18 through to 65% for 94 patients within Cluster 24. A further striking feature was that values obtained from the training cohort were strongly predictive of outcomes in the validation cohorts. As such, these findings show clustering of demographic, clinical, and laboratory features taken at the time of hospital entry is predictive of medium-term mortality. This is important as this information could be used to guide appropriate triage and clinical management at an early stage of the patient journey.

It was interesting to compare the mortality rates for patients within wave one and wave two of the pandemic treated at the same hospital. Dexamethasone emerged as a standard of care for all patients with severe COVID-19 who required oxygen therapy in July 2020 and as such would have been a default management approach for such patients in wave 2. The overall mortality rate fell by nearly 50% during this period and lower 28-day mortality rates were seen for many clusters within wave 2, although no clear improvement was seen in the very high-risk clusters of 24, 4, and 17 which included many patients with cancer, dementia, and cardiovascular disease. This reveals that cluster analysis could be of value in assessing the differential impact of new therapies on patient groups and identifying those which represent an unmet need.

Furthermore, this use of an unsupervised machine learning approach to define patient subgroups enables identification of new and potentially unexpected interactions between clinical and laboratory variables. Patients in high-risk groups were characterized by medical comorbidity in association with laboratory variables such as impaired renal function and metabolic acidosis. The interaction between comorbidities within clusters also revealed a number of interesting features (Figure 8). For example, although patients with dementia and cardiovascular disease were enriched within the high-risk Cluster 24, this combination was also seen in several lower risk groups such as 15, 9, 19, and 29. As such, a striking feature from the analysis was that clusters comprised complex combinations of clinical determinants that would not have been easily predictable from clinical assessment. Ethnicity data was not available for the training cohort and in the validation cohorts >50% were of white ethnicity but this was not a key driver of clustering with only Cluster 15 associated with white ethnicity.

In summary, the application of untargeted machine learning to clinical data collected routinely at hospital entry allowed identification of subpopulations of COVID-19 patients with distinct mortality rates and clinical presentation on the day of admission. These were consistent across different clinical datasets and uncovered prognostic interactions between clinical variables that could influence management decisions. Furthermore, such clusters may also reveal activation of different biological pathways and might therefore uncover new mechanisms of COVID-19 susceptibility and a means to stratify therapeutic interventions in phenotype-informed randomized control trials, such as optimized management of renal impairment, metabolic acidosis, and cardiovascular disease for patients in Cluster 24. This approach has application to a wide variety of clinical conditions and contributes to the growing expectation that artificial intelligence systems will transform healthcare delivery and operate in real time to support clinical decision making in both acute and chronic conditions.

### Limitations of the study

Potential limitations of our study include the fact that the training cohort was derived from several clinical sites and could contain site-specific effects with more exposure to reporting errors. The use of unconstrained covariance matrices in model fitting may limit scalability to larger and more complex datasets. In this context, a diagonal covariance matrix may be more appropriate. In addition, missing information was apparent across the three cohorts (Figure S1).

## STAR★Methods

### Key resources table

**Table:** 

REAGENT or RESOURCE	SOURCE	IDENTIFIER
**Deposited data**		

Development data	Geriatric Medicine Research Collaborative (https://www.gemresearchuk.com/, gemresearch.uk@gmail.com).	N/A
Validation data	PIONEER Health Data Research Hub (https://www.pioneerdatahub.co.uk/, pio-neer@uhb.nhs.uk).	N/A

**Software and algorithms**		

R: A language and environment for statistical computing	R Core Team (2019)	https://www.R-project.org/
RStudio	RStudio Team (2016)	http://www.rstudio.com/
mice (R package)	Van Buuren and Groothuis-Oudshoorn (2011)	https://CRAN.R-project.org/package=mice
FactoMineR (R package)	Le et al., (2008)	https://CRAN.R-project.org/package=FactoMineR
factoextra (R package)	Kassambara and Mundt (2020)	https://CRAN.R-project.org/package=factoextra
intrinsicDimension (R package)	Johnsson (2019)	https://CRAN.R-project.org/package=intrinsicDimension
uwot (R package)	Melville (2020)	https://CRAN.R-project.org/package=uwot
mclust (R package)	Scrucca et al. (2016)	https://CRAN.R-project.org/package=mclust
cluster (R package)	Maechler et al. (2019).	https://CRAN.R-project.org/package=cluster
forcats (R package)	Wickham, 2021	https://CRAN.R-project.org/package=forcats
DataCombine (R package)	Gandrud, 2016	https://CRAN.R-project.org/package=DataCombine
confintr (R package)	Mayer (2020)	https://CRAN.R-project.org/package=confintr
sjstats (R package)	Lüdecke, 2021	https://CRAN.R-project.org/package=sjstats
dplyr (R package)	Wickham et al., 2021	https://CRAN.R-project.org/package=dplyr
reshape2 (R package)	Wickham, 2007	http://www.jstatsoft.org/v21/i12/
ggplot2 (R package)	Wickham (2016)	https://CRAN.R-project.org/package=ggplot2
Code from this paper	GitHub: https://doi.org/10.5281/zenodo.6320265	https://github.com/wdsquared/UMAP-assisted-clustering

### Resource availability

#### Lead contact

Further information and requests for resources should be directed to and will be fulfilled by the lead contact, Paul Moss (p.moss@bham.ac.uk).

#### Materials availability

No materials were used in this study.

### Method details

#### Patient population

Clinical data were acquired for patients admitted to hospital with COVID-19. The CovidCollab cohort is a multicentre dataset of 6099 patients admitted between January and August 2020 and was used as the training cohort in model development(Alsahab et al., 2021). Two cohorts of patients admitted to the University Hospitals NHS Foundation Trust Birmingham (UHBFT) were used for validation. The first cohort (‘wave 1’) includes 996 patients admitted between January and August 2020. The second cohort (‘wave 2’) includes 1011 patients admitted between September 2020 and January 2021 (Table 1). Patients tested positive for SARS-CoV-2 by PCR and/or antibody test within 14 days of admission. Mortality at day 28 (including after discharge) was determined for each patient.

Ethical approval was provided by the East Midlands–Derby REC (reference: 20/EM/0158) for the PIONEER Research Database (data from University Hospitals Birmingham). For CovidCollab data, local, regional and national approvals were obtained from all participating sites. In the UK, this study was registered as clinical audit or service evaluation, with approval granted in line with local information governance policies, in line with assessment and guidance by the Health Research Authority. At the lead site (University Hospitals Birmingham NHS Trust), this study was registered as clinical audit (CARMS-15986). In other countries, local principal investigators were responsible for obtaining approvals in line with their local, regional and national guidelines and recommendations. Only routinely collected data were collected and patient care was not altered by this study. Anonymised data were securely transferred to the Birmingham Centre for Prospective and Observational Studies, University of Birmingham via REDCap. All sites were required to confirm that approvals were in place prior to being provided with logins; written data sharing agreements were arranged where requested by individual sites.

#### Variables and missing data

Data obtained for each patient included demographic, clinical and laboratory variables taken on the day of admission (Tables 1 and S1). Analysis was conducted in R (R Core Team, 2019) with RStudio (RStudio Team, 2016). Missing data were quantified by cohort (Figure S1) and imputed from the observed data by predictive mean matching (Bailey et al., 2020; De Silva et al., 2019; Morris et al., 2014). The Multivariate Imputation by Chained Equations (MICE) (Van Buuren and Groothuis-Oudshoorn, 2011) algorithm was applied independently to each cohort under fully conditional specification. 28 variables (continuous and binary) (Table 1) were used for model development for which the maximum proportion of missing observations was 36% in the training cohort, 43% in wave 1 and 42% in wave 2. A single imputation of the training cohort was used for model development. Multiple imputations of waves 1 and 2 were analysed for validation (n. imputations = 5).

#### Data processing

UMAP was applied to the training cohort prior to model development (Melville, 2020) (Figure S2). Binary variables were one-hot encoded (0/1), and all variables standardised prior to transformation (mean 0, variance 1). UMAP transformed the 28 clinical variables onto a lower dimensional embedding. Initial hyper-parameters were selected by visualising a 2-D embedding output (Figure S3). A Euclidean distance metric was used with a target of 40 nearest neighbours and a minimum distance of 0.25.

#### Clustering model development

Gaussian mixture model (GMM) clustering was applied to automatically label the distribution of patients after transformation by UMAP (Rasmussen and Ghahramani, 2001; Scrucca et al., 2016) (Figure S2). An Expectation-Maximization (EM) algorithm was used to estimate GMM parameters with unconstrained covariance matrices (Scrucca et al., 2016).

The number of UMAP dimensions to output for clustering, *D*, was determined qualitatively. Maximum likelihood estimation of intrinsic dimension (Johnsson, 2016, 2019; Levina and Bickel, 2004) and Factor Analysis of Mixed Data (FAMD) (Bécue-Bertaut and Pagès, 2008; Kassambara and Mundt, 2020; Le et al., 2008) were applied to determine a range for D based on data complexity. Iterative UMAP dimension reduction was used to test the effect of D on the clustering model. GMMs were fitted to each D-dimensional embedding with between 2 and 50 mixture components. Average silhouette width (Batool and Hennig, 2021; Maechler et al., 2019; Rousseeuw, 1987) and Bayesian information criterion (BIC) (Gideon, 1978; Scrucca et al., 2016) were measured. The maximum D was selected which did not substantially reduce silhouette width or result in high variability in BIC between models. From this, 4 dimensions (4-D embedding) were selected for onward analysis (Figure S4).

The number of mixture components, *K*, and therefore clusters of patients, was determined using the optimal BIC, maximum silhouette width, and a qualitative assessment of BIC and silhouette width plots (manual BIC, manual silhouette). If multiple values for K were selected, the diversity of mortality rates was then compared between these models and the mortality rate at day 28 after hospital admission calculated for K clusters. ISARIC4C modelling had shown that mortality rates varied markedly between high risk and low risk groups (Figure 1) and the model with the largest range of mortality rate was therefore retained. Based on this, the final reported model was a GMM fitted with K = 29 (Figure S4).

#### Clustering validation data

A two-stage process assigned patients from waves 1 and 2 into clusters detected by the GMM in model development. First, the UMAP transformation learned from the training cohort was applied to embed the validation cohorts onto the same 4-D embedding. Secondly, the trained GMM was applied to predict which clusters the new observations should be assigned to with the highest probability. This was repeated for 5 imputations and a majority vote was taken as the final cluster classification (Basagana et al., 2013). Separation of clusters was assessed for each imputation by silhouette analysis and estimates were pooled with a 95% confidence interval (CI) by applying Rubin’s rules (Marshall et al., 2009).

#### 2-D visualisation

For visualisation purposes the UMAP analysis was repeated to transform the 4-D embedding of the training cohort from 4-D to 2-D. The same transformation was applied to each validation cohort and scatter plots (UMAP plots) were created (Wickham, 2016).

### Quantification and statistical analysis

#### Comparing cohorts

Complete cases of each variable were compared between the training and validation cohorts using a Wilcoxon test for continuous variables or Fisher’s test for categorical variables (Fisher, 1935; Rey and Neuhäuser, 2011). The Benjamini-Hochberg procedure was applied to adjust for multiple testing (Benjamini and Hochberg, 1995). Mortality rate at day 28 after hospital admission was calculated for individual clusters across each of the three patient cohorts and odds-ratios were calculated (95% CI, 1000 bootstrap resamples) (Mayer, 2020).

#### Cluster characterisation analysis

Fisher’s tests were applied to detect variables significantly associated with each cluster (p = 0.001). Continuous variables were categorised into ordinal factors from literature review and tests applied to each factor level (Table S2). Only variables where the association was significant in the training cohort and at least one validation cohort were reported here. Results were summarised using word clouds with variables scaled by the strength of the association in the training cohort (Chen et al., 2010; Fellows, 2018; Le Pennec and Slowikowski, 2019). Word clouds were overlaid on to a UMAP plot of the training cohort to aid interpretation. Clusters with fewer than 10 patients in either validation cohort were excluded.

#### Risk classification

ISARIC4C mortality score was calculated for each patient (Knight et al., 2020) and revealed 0–88% risk of death within 28 days. Scores were divided into low (0–3), intermediate (4–8), high (9–14) and very high risk (15–21) for plotting purposes. The training cohort was assigned a score based off a single imputation, waves 1 and 2 were assigned a score based off the mode across 5 imputations. Kaplan–Meier (KM) (Therneau and Grambsch, 2000) curves were constructed to estimate survival rates with 95% CI. A log-rank test was applied to test for differences between groups.

#### Role of the funding source

The work was funded from an NIHR grant to PM. The sponsor of the ethics had no role in decision to publish, collection of data or authorship. The contributions by NA, ES, KN, MP, CS and TT were funded by the Medical Research Council UK Research and Innovation (reference COV0306) during the study. The funder had no role in developing the research question or the study protocol.

## Data Availability

• The clinical data reported in this study cannot be deposited in a public repository because of ethical constraints. To request access to the development data, contact the Geriatric Medicine Research Collaborative (https://www.gemresearchuk.com/, gemresearch.uk@gmail.com). To request access to the validation data, contact the PIONEER Health Data Research Hub (https://www.pioneerdatahub.co.uk/, pioneer@uhb.nhs.uk). • All original code has been deposited on GitHub (GitHub: https://github.com/wdsquared/UMAP-assisted-clustering) and is publicly available as of the date of publication. DOIs are listed in the key resources table. • Any additional information required to reanalyse the data reported in this paper is available from the lead contact upon request.
