# Supplementary material for: Machine learning of COVID-19 clinical data identifies population structures with therapeutic potential
Source: iScience. 2022 May 31;25(7):104480. doi: 10.1016/j.isci.2022.104480 (PMC9153184; doi:10.1016/j.isci.2022.104480)
Supplement: Document S1. Figures S1–S6, Tables S1 and S2 [file mmc1.pdf]

iScience, Volume *25*

Supplemental information

Machine learning of COVID-19 clinical data identifies population structures with therapeutic potential

David Greenwood, Thomas Taverner, Nicola J. Adderley, Malcolm James Price, Krishna Gokhale, Christopher Sainsbury, Suzy Gallier, Carly Welch, Elizabeth Sapey, Duncan Murray, Hilary Fanning, Simon Ball, Krishnarajah Nirantharakumar, Wayne Croft, and Paul Moss

Supplemental information

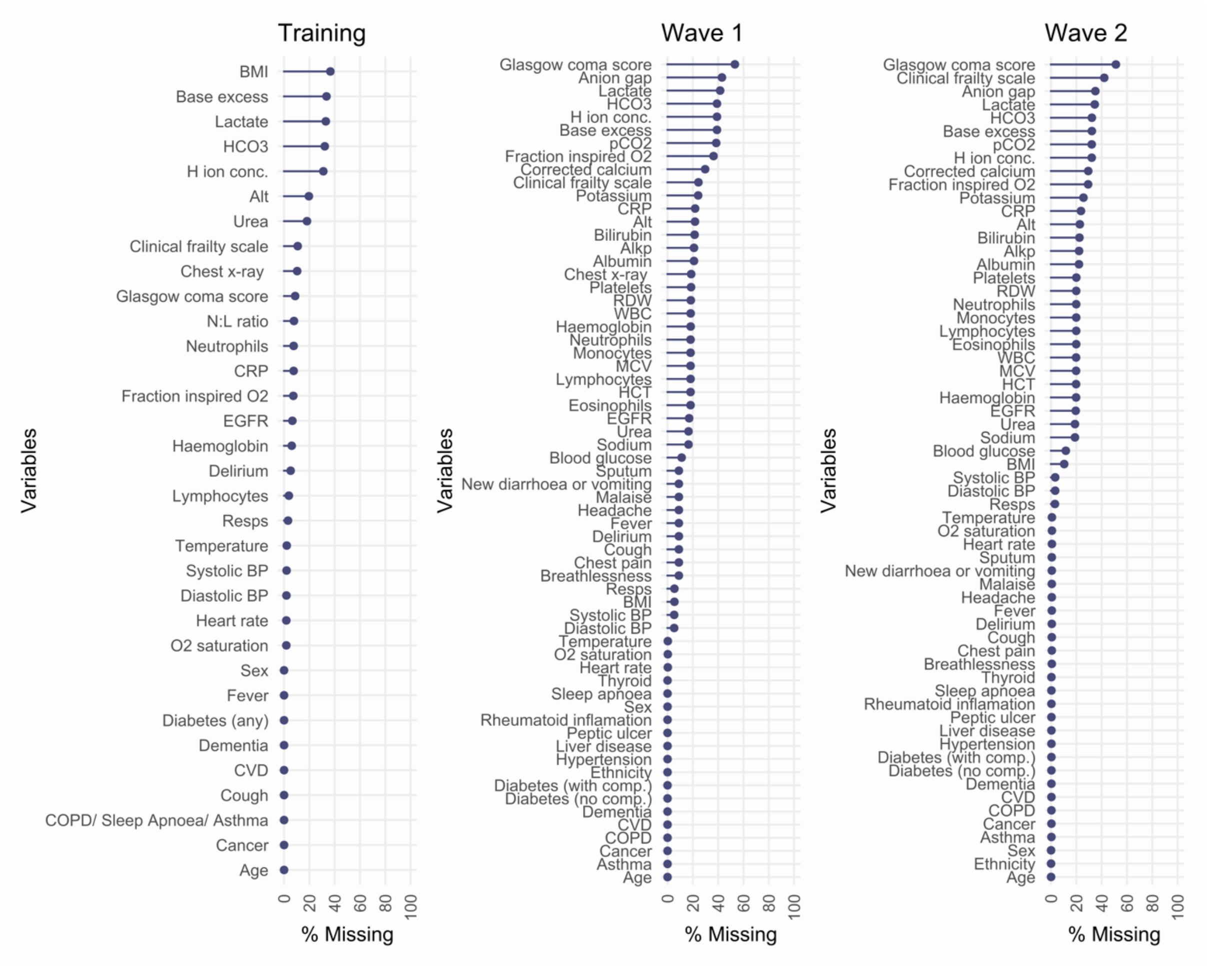

Figure S1 Percentage of missing observations. Related to Figure 2

The percentage of missing observations for each variable in the training, wave 1 and wave 2 cohorts.

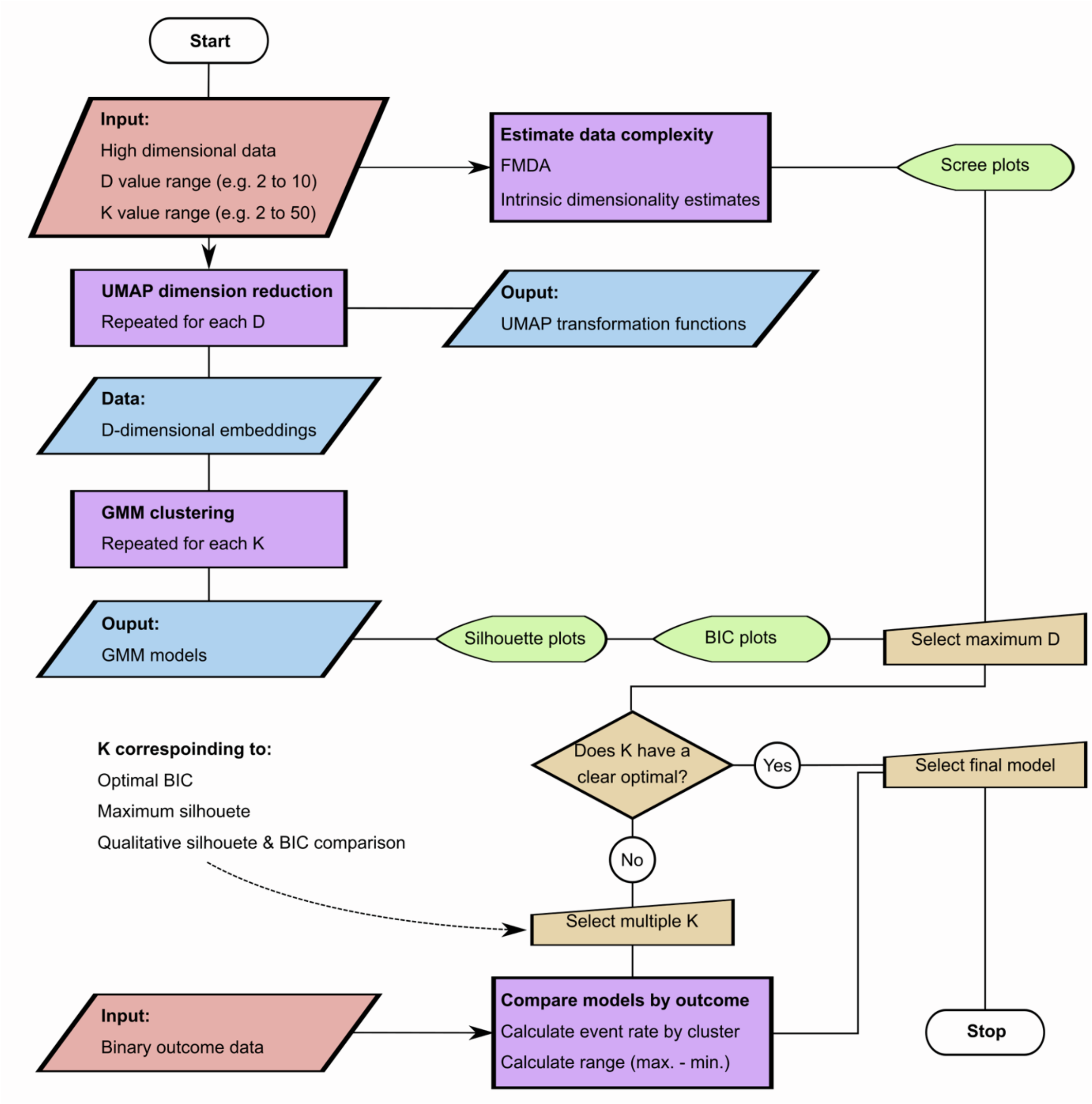

Figure S2 Workflow for clustering model development. Related to Figure 2

A clustering model was developed by training a GMM with UMAP dimension reduction as a pre-processing step. The number of dimensions, D, to embed the data and the number of mixture components, K, for GMM fitting were determined as follows. Data complexity as measured by intrinsic dimensionality estimation and FAMD was used to select a range for D. UMAP was applied iteratively with each value of D, and models were fitted to each embedding. The maximum D was selected which did not substantially reduce silhouette width or result in high variability in BIC between models. Possible values of K were selected based on BIC and silhouette width. If both methods indicated a similar K, model development was complete. If multiple values for K were selected, the diversity of mortality rates was compared between models. Mortality rate at day 28 after hospital admission was calculated for K clusters. The model with the largest range (maximum-minimum) was retained.

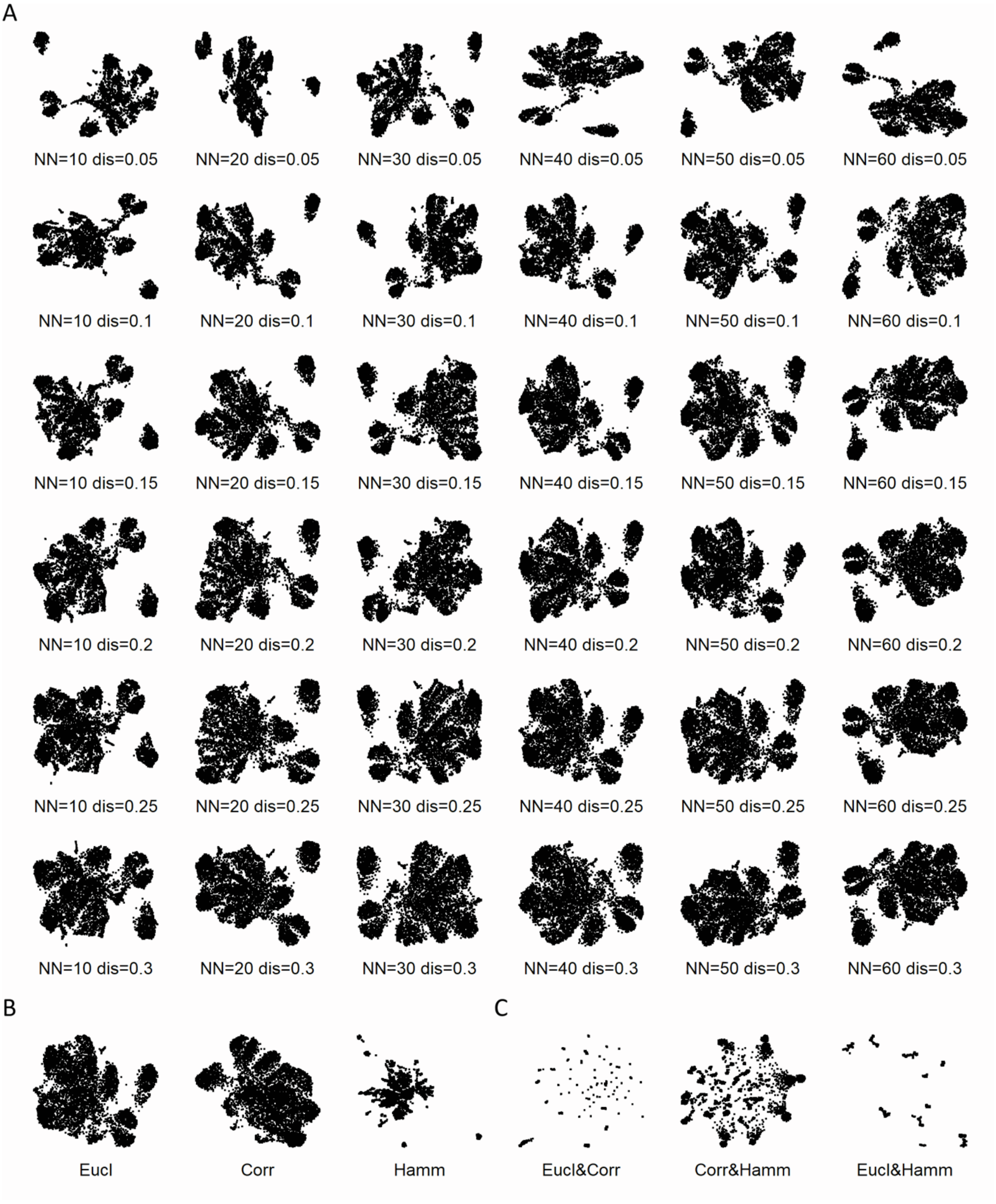

Figure S3 Selection of UMAP hyper-parameters. Related to Figure 2

- **(A)** UMAP hyper-parameters, nearest neighbours (NN) = 40 and minimum distance (dis) = 0.25 were selected by visualising a 2-D embedding of the Training cohort using a Euclidean distance metric (Eucl).
- **(B)** Additional distance metrics, Hamming (Hamm) and Pearson correlation (Corr) were also compared, the examples shown used NN=45 and dis=0.25.
- **(C)** Combinations of metrics, Eucl/Corr for continuous data with Corr/Hamm were also tested with NN=45 and dis=0.25. However, this would prevent the use of a UMAP transformation with new observations so was deemed unsuitable for model development purposes.

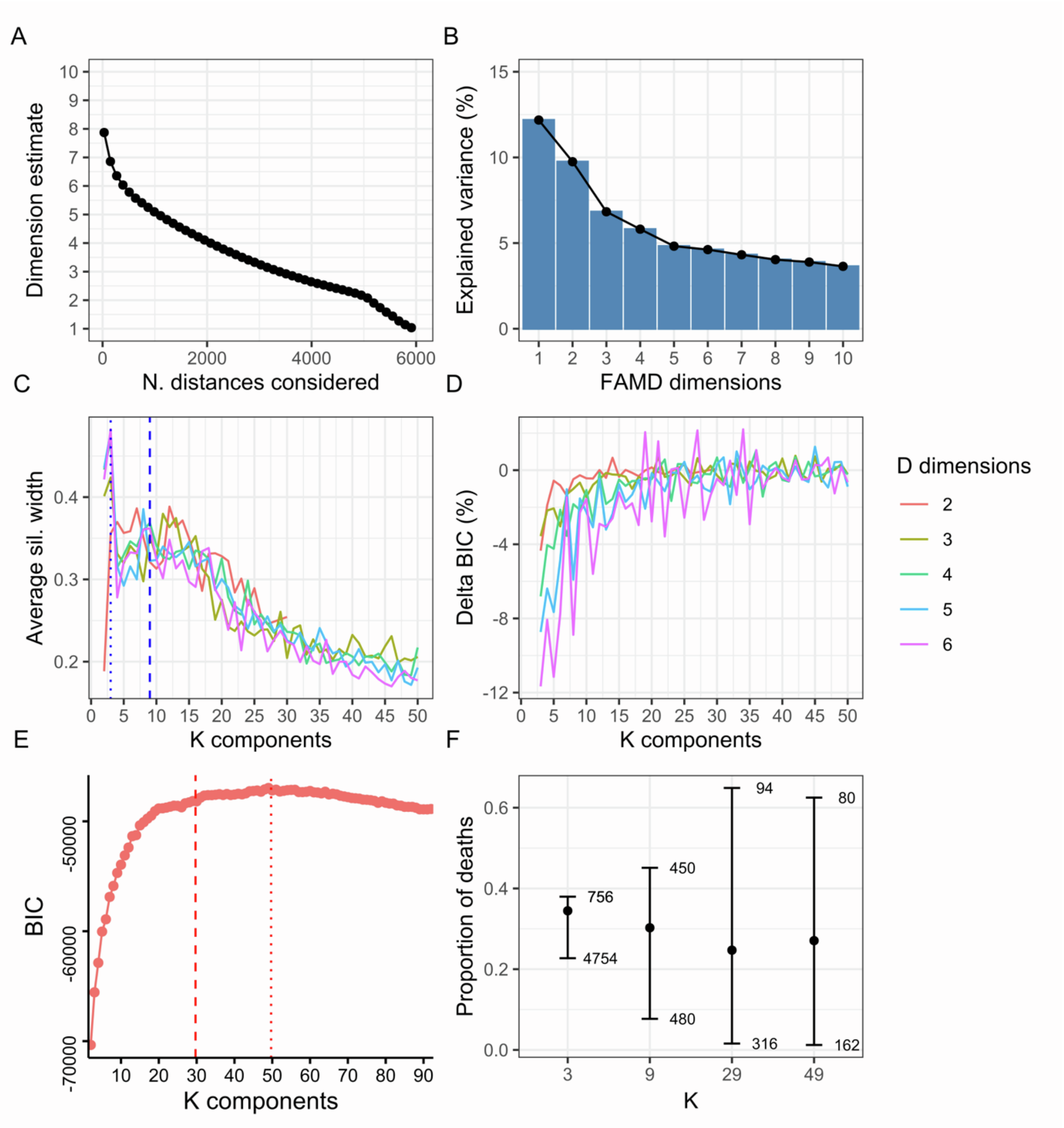

Figure S4 Selection of the number of UMAP dimensions and GMM components based on the training cohort. Related to Figure 3.

- **(A)** Intrinsic dimension estimation by number (N.) distances measured, estimated globally.
- **(B)** FAMD scree plot of the percentage of explained variance by eigenvector.
- **(C)** Average silhouette (sil.) width by number of components in the GMM (K), grouped by the number of UMAP dimensions used in model fitting (D). Dotted and dashed lines indicate K selected by maximum sil. width (K=3) and manually by qualitative assessment (K=9).
- **(D)** Change in BIC (delta BIC) with increasing K, grouped by D.
- **(E)** BIC by K for a 4-D embedding. Dotted and dashed lines indicate K selected by optimal BIC (K=49) and manually by qualitative assessment (K=29).
- **(F)** Maximum, median and minimum proportion of deaths by cluster for selected values of K on a 4-D embedding. Annotated by number of patients in the cluster.

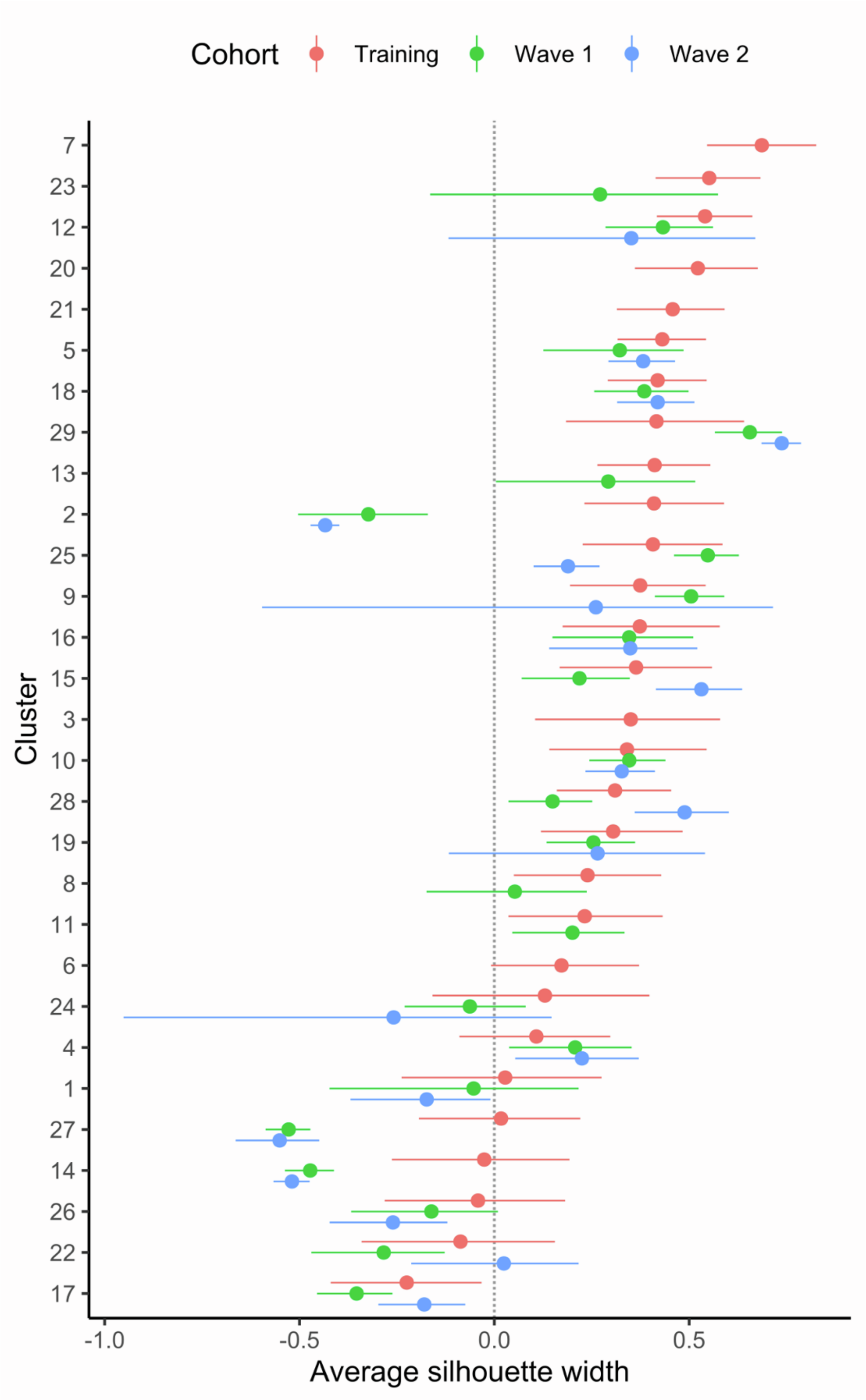

Figure S5 Cluster separation by silhouette width. Related to Figure 3

Average cluster silhouette width, a measure of cluster separation, defined for each cluster. This measures how similar observations are within a cluster compared to the most similar cluster, with higher values suggesting better clustering configuration. Single imputation testing was used for the training cohort whilst validation cohorts were tested across 5 imputations and a single estimate was pooled (mean, 95% CI).

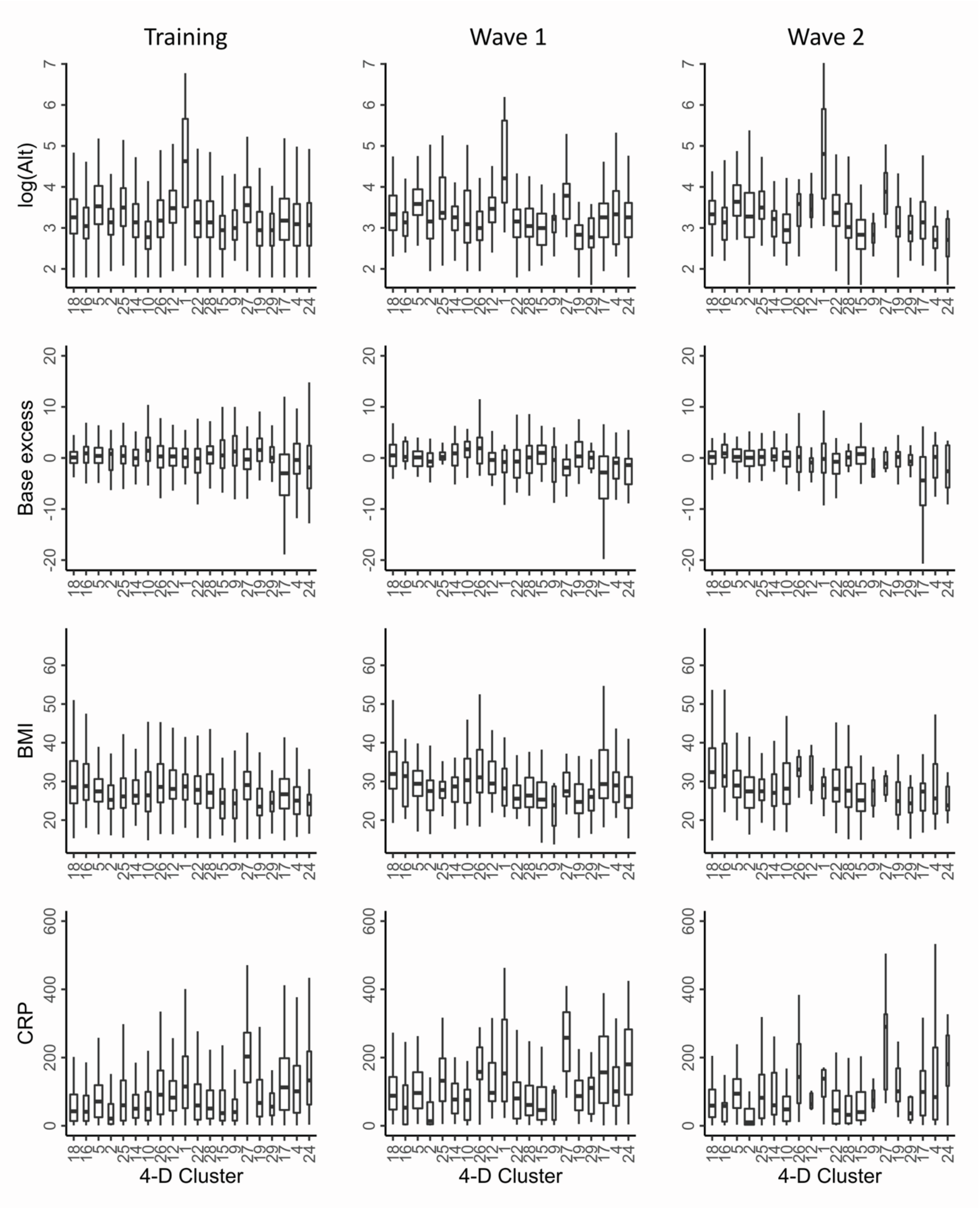

Training
Wave 1
Wave 2
log(Alt)
Base excess
BMI
CRP
4-D Cluster
4-D Cluster
4-D Cluster

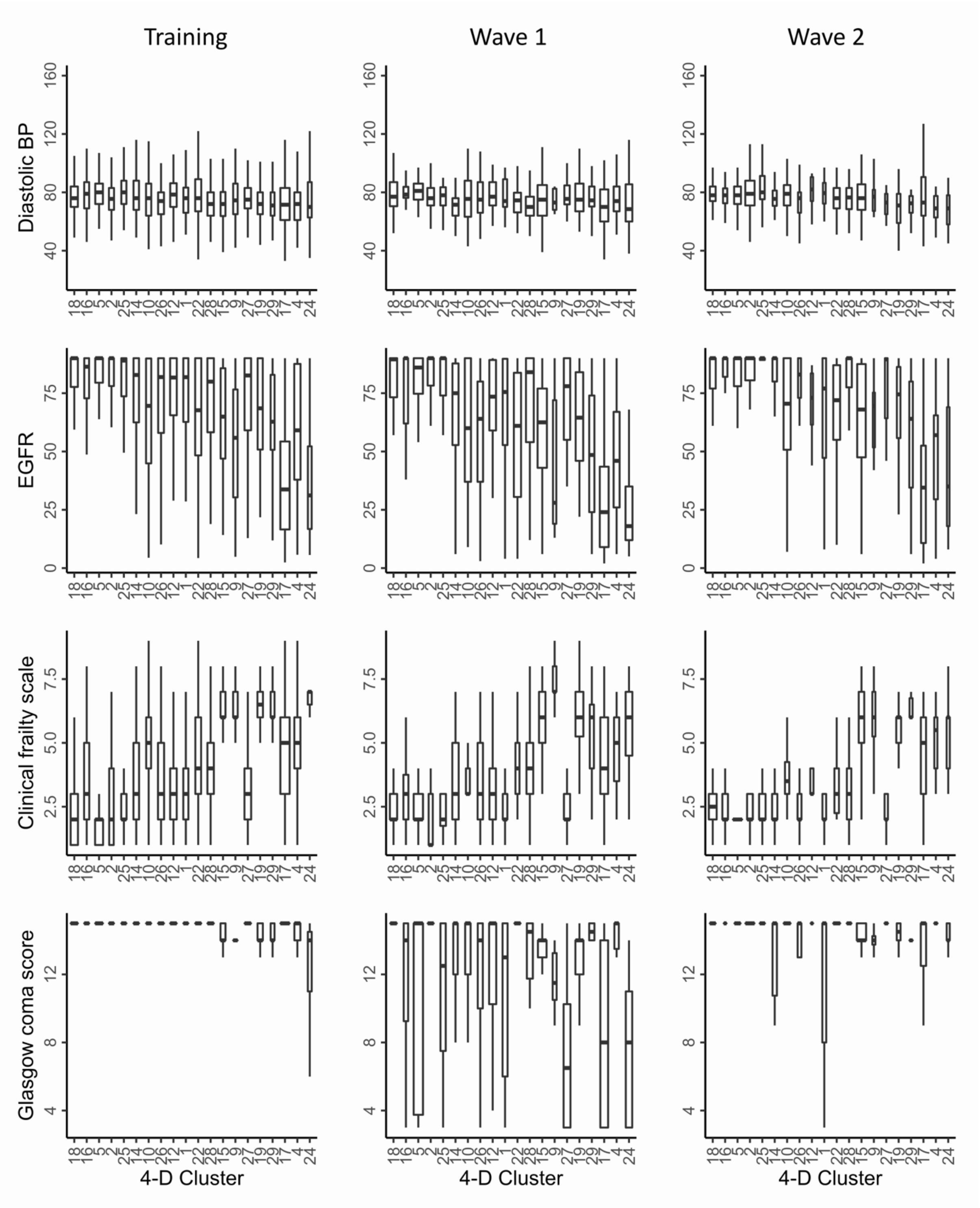

Training
Wave 1
Wave 2
Diastolic BP
EGFR
Clinical frailty scale
Glasgow coma score
4-D Cluster
4-D Cluster
4-D Cluster

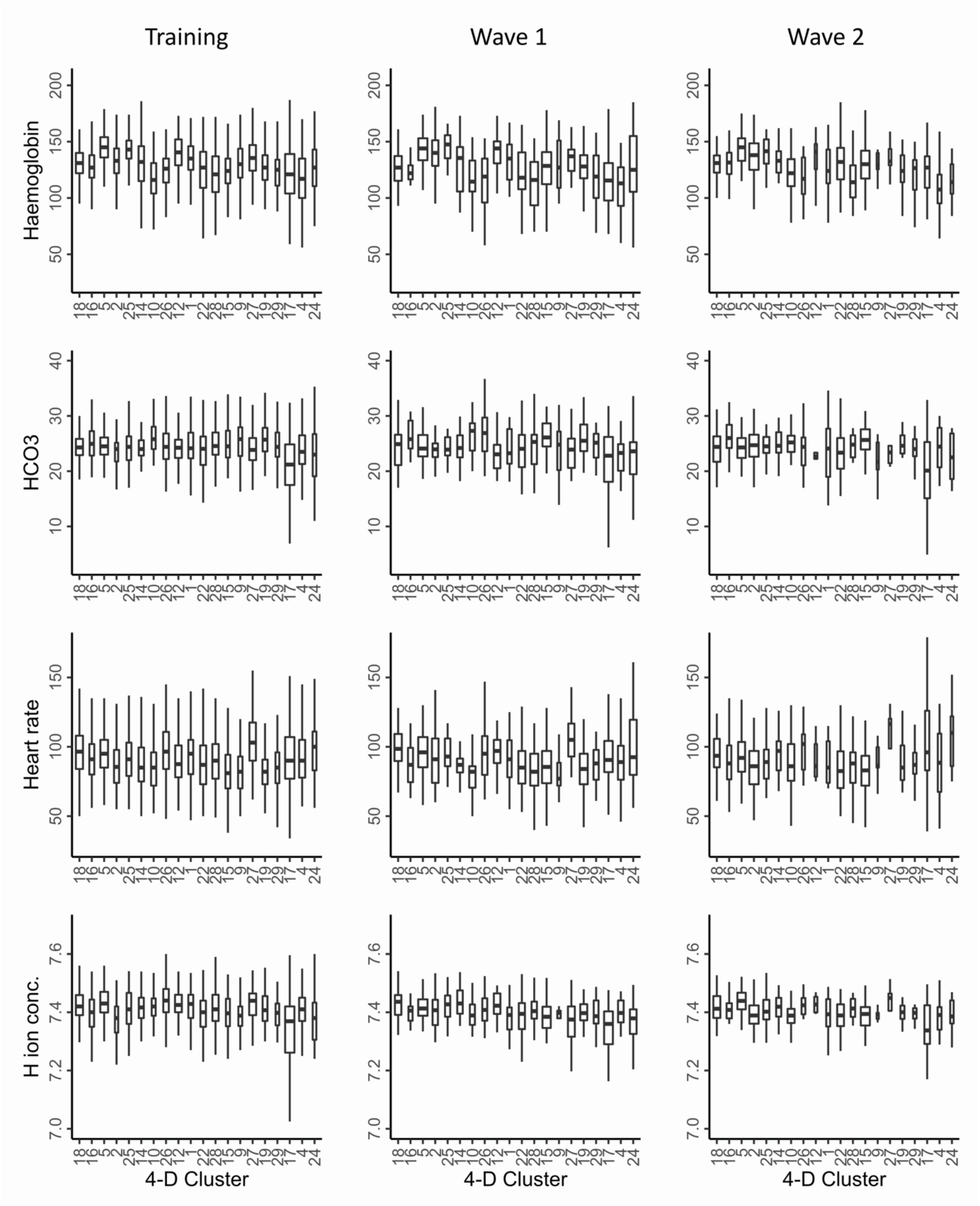
Training
Wave 1
Wave 2
Haemoglobin
HCO3
Heart rate
H ion conc.
4-D Cluster
4-D Cluster
4-D Cluster
18 16 5 2 25 14 10 26 12 1 22 28 15 9 27 19 29 17 4 24

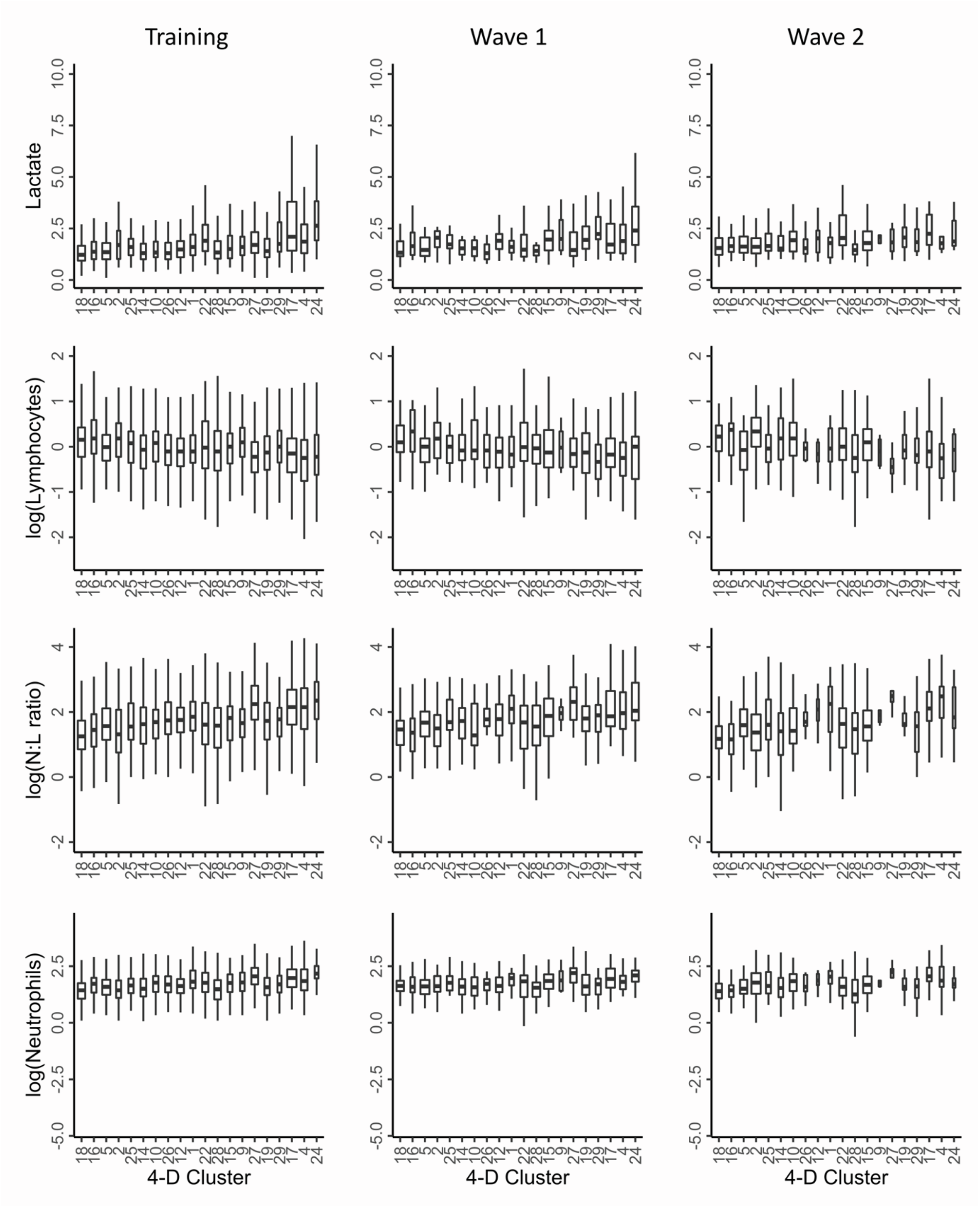

Training
Wave 1
Wave 2
Lactate
log(Lymphocytes)
log(N:L ratio)
log(Neutrophils)
4-D Cluster
4-D Cluster
4-D Cluster

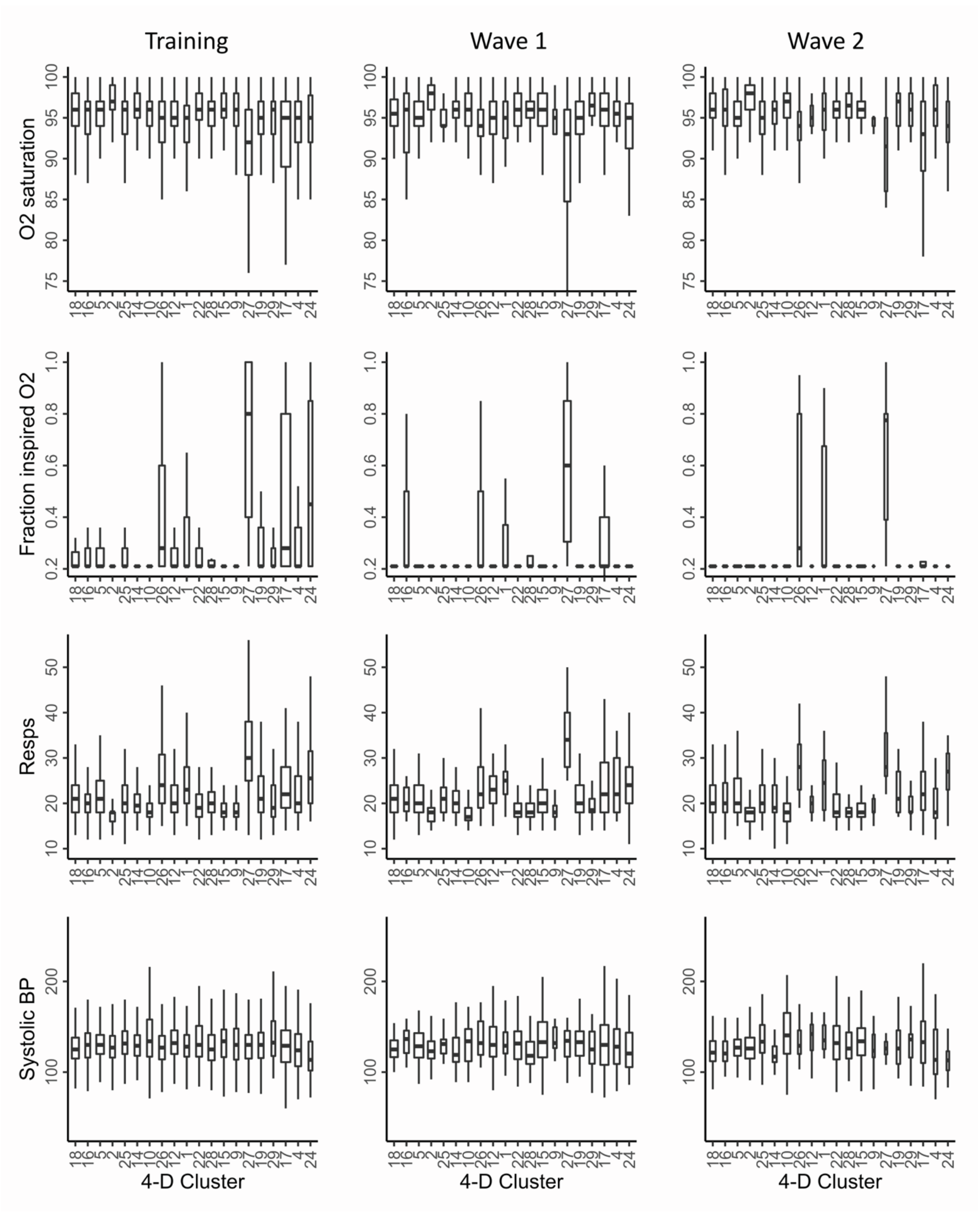
Training
Wave 1
Wave 2
O2 saturation
Fraction inspired O2
Resps
Systolic BP
18 16 5 2 25 14 10 26 12 1 22 28 15 9 27 19 29 17 4 24
4-D Cluster
4-D Cluster
4-D Cluster

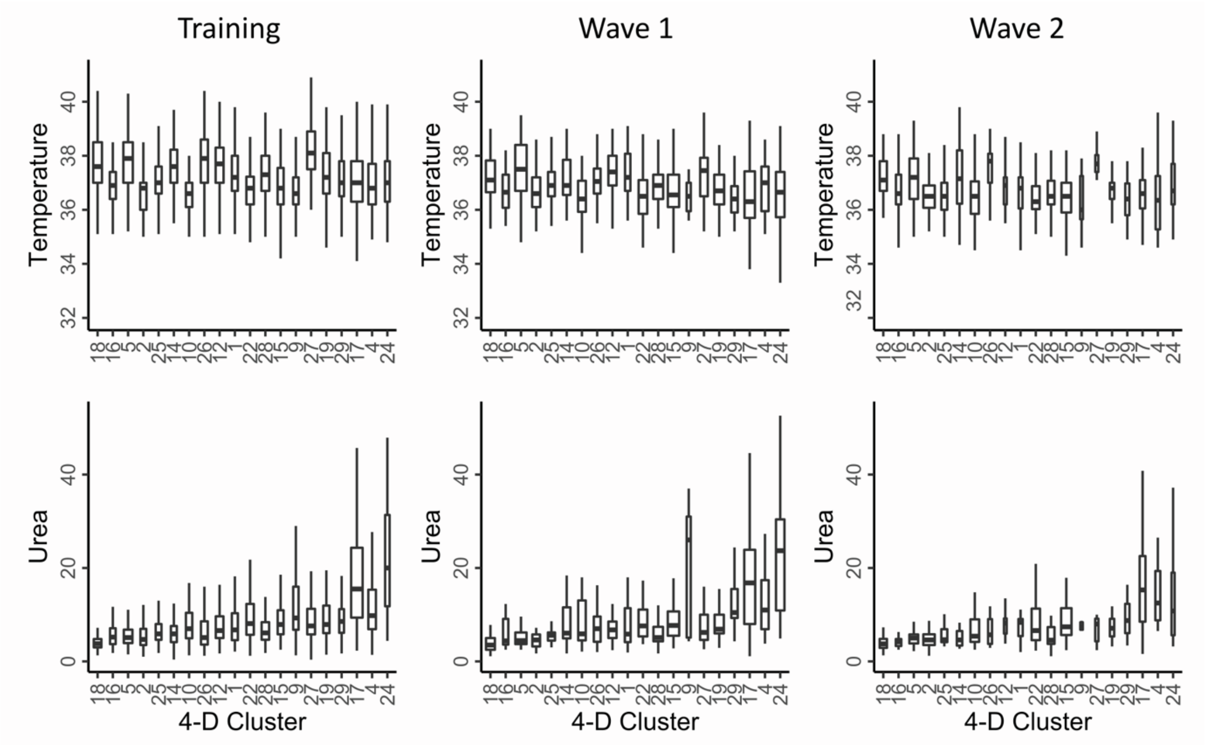

Figure S6 Variable distributions by cluster. Related to Figures 6-8

Distribution of clinical variables by cluster across the three patient cohorts. Boxes represent the median and IQR with whiskers extended to the lowest or highest data within 1.5 IQR of the lower or upper quartiles. Clusters are ordered along the x-axis in relation to increasing rate of associated 28 day mortality. Missing observations were excluded, and the number of observations was used to scale the width of each box. Analysis was performed on clusters which comprised at least 10 patients in each cohort.

Supplementary Tables

Supplementary Table 1 Additional variables within validation cohorts summarised by median (continuous) or count (categorical). Related to Figure 2

	Cohort	
**Variable**	**Wave 1**	**Wave 2**
N patients	996	1011
Age (years) [IQR]	70.00 [56.00, 83.00]	64.00 [50.00, 79.00]
Male (%)	564 (57)	525 (52)
Ethnicity (%)		
Black	63 (6)	45 (4)
Other	214 (21)	189 (19)
South Asian	161 (16)	267 (26)
White	558 (56)	510 (50)
Liver disease (%)	64 (6)	81 (8)
COPD (%)	269 (27)	257 (25)
Asthma (%)	157 (16)	183 (18)
Breathlessness (%)	551 (61)	429 (43)
Chest pain (%)	39 (4)	65 (6)
Diabetes (no comp.) (%)	250 (25)	270 (27)
Diabetes (with comp.) (%)	106 (11)	48 (5)
Headache (%)	42 (5)	48 (5)
Hypertension (%)	628 (63)	519 (51)
Malaise (%)	183 (20)	171 (17)
New diarrhoea or vomiting (%)	55 (6)	70 (7)
Peptic ulcer (%)	26 (3)	21 (2)
Rheumatoid inflammation (%)	49 (5)	42 (4)
Sleep apnoea (%)	47 (5)	34 (3)
Sputum (%)	84 (9)	72 (7)
Thyroid (%)	102 (10)	82 (8)
Alkaline phosphatase (U/L) [IQR]	81.00 [63.00, 112.00]	82.00 [65.00, 107.00]
Anion gap (mmol/L) [IQR]	19.20 [17.20, 21.40]	19.20 [17.70, 20.90]
Bilirubin (umol/L) [IQR]	11.00 [8.00, 16.00]	10.00 [7.00, 14.00]
Blood glucose (mmol/L) [IQR]	7.14 [6.09, 9.09]	6.90 [5.85, 8.80]
Corrected calcium (mmol/L) [IQR]	2.32 [2.24, 2.42]	2.29 [2.21, 2.37]
Eosinophils (10*9/L) [IQR]	0.01 [0.00, 0.06]	0.02 [0.00, 0.08]
HCT (L/L) [IQR]	0.39 [0.34, 0.43]	0.39 [0.35, 0.42]
MCV (fL) [IQR]	88.80 [84.15, 93.20]	86.50 [82.20, 90.70]
Monocytes (10*9/L) [IQR]	0.51 [0.33, 0.72]	0.53 [0.35, 0.76]
Neutrophils (10*9/L) [IQR]	5.80 [4.05, 8.35]	5.20 [3.60, 7.97]
Platelets (10*9/L) [IQR]	214.00 [164.50, 283.00]	235.00 [180.00, 305.00]
Potassium (mmol/L) [IQR]	4.10 [3.80, 4.50]	4.10 [3.80, 4.50]
RDW (%) [IQR]	13.80 [12.80, 15.20]	13.60 [12.70, 14.80]
Sodium (mmol/L) [IQR]	138.00 [135.00, 141.00]	137.00 [135.00, 140.00]
WBC (10*9/L) [IQR]	7.50 [5.60, 10.30]	7.20 [5.30, 10.20]

Comp., complications; COPD, chronic obstructive pulmonary disease; fL, femtolitre; HCT, haematocrit; IQR, interquartile range; L, Litre; MCV, mean corpuscular volume; min., minute; mmol,

Millimoles; N, number; RDW, red cell distribution width; U, units; umol, micromole; WBC, White blood cell count

Supplementary Table 2 Thresholds and factor labels. Related to Figure 2.

Variable	Breaks / Levels	Factor labels
Age	0, 30, 40, 50, 60, 70, 80, 90, Inf	
Alanine Aminotransferase	0, 55, 100, 8000	↓↓,↕,↑↑
Albumin	0, 25, 35, 8000	↓↓,↕,↑↑
Alkaline Phosphatase	0, 130, 8000	↓↓,↑↑
Anion Gap	0, 6, 16, 8000	↓↓,↕,↑↑
Asthma		
Base Excess	-50, -2, 2.01, 8000	↓↓,↕,↑↑
Bilirubin	0, 21, 8000	↓↓,↑↑
Blood Glucose	0, 7.8, 8.5, 8000	↓↓,↕,↑↑
BMI	-Inf, 18.5, 25, 30, Inf	↓↓,↓,↑,↑↑
Breathlessness		
Cancer (any malignancy)		
Cardiovascular disease		
Chest pain		
Chest x-ray	Appears clear, Local consolidation, Ground glass opacity / Bilateral infiltrates	Clear x-ray, Local consol. x-ray, GGO/bilat. x-ray
Clinical frailty scale	0, 3, 6, 10	↓↓,↕,↑↑
COPD		
Corrected calcium	0, 2.2, 2.6, 8000	↓↓,↕,↑↑
Cough		
CRP	0, 10, 100, 8000	↓↓,↕,↑↑
Delirium		
Dementia		
Diabetes (no comp.)		
Diabetes (with comp.)		
Diastolic BP	0, 90, 8000	↓↓,↑↑
Eosinophils	0, 0.405, 8000	↓↓,↑↑
EGFR	-Inf, 30, 59, 89, Inf	↓↓,↓,↑,↑↑
Fever		
Glasgow coma score	3, 15, 8000	↓↓,↑↑
Haemoglobin	0, 115, 154, 8000	↓↓,↕,↑↑
HCO3	0, 22, 29, 8000	↓↓,↕,↑↑
HCT	0, 0.5, 8000	↓↓,↑↑
Headache		
Heart Rate	0, 80, 100, 8000	↓↓,↕,↑↑
Hydrogen ion conc.	0, 7.299999, 7.350001, 7.450001, 8000	↓↓,↓,↑,↑↑
Hypertension		
Lactate	0, 2.21, 8000	↓↓,↑↑

Liver disease		
Lymphocytes	0, 1.5, 8000	↓↓,↑↑
Malaise		
MCV	0, 80, 96, 8000	↓↓,↕,↑↑
Monocytes	0, 0.2, 0.81, 8000	↓↓,↕,↑↑
Neutrophil:Lymphocyte ratio	0, 2.21, 4.83, 8000	↓↓,↕,↑↑
New diarrhoea or vomiting		
O2 saturation	0, 80, 89, 94, 8000	↓↓,↓,↑,↑↑
pCO2	0, 4.67, 6.4, 8000	↓↓,↕,↑↑
Peptic ulcer		
Platelets	0, 150, 400, 8000	↓↓,↕,↑↑
pocFiO2	-Inf, 0.28, 0.5, Inf	↓↓,↕,↑↑
Potassium	0, 2.5, 5.3, 8000	↓↓,↕,↑↑
RDW	0, 11.5, 15.5, 8000	↓↓,↕,↑↑
Respiratory rate	0, 20, 8000	↓↓,↑↑
Rheumatoid inflammation		
Sex		
Sleep apnoea		
Sodium	0, 133, 145, 8000	↓↓,↕,↑↑
Sputum		
Systolic BP	0, 140, 8000	↓↓,↑↑
Temperature	0, 37.8, 8000	↓↓,↑↑
Thyroid		
Urea	0, 7.8, 8000	↓↓,↑↑
WBC	0, 3.9, 10.9, 8000	↓↓,↕,↑↑

BP, blood pressure; Comp., complications; conc., concentration; CRP, C-reactive protein; pCO2, partial pressure of carbon dioxide; pocFiO2, fraction of in-spired O2; RDW, red cell distribution width; WBC, White blood cell count
